# Zero-Dimensional Bismuth Halide Thin Films with High Orientation and Broadband Emission

**DOI:** 10.1021/acs.inorgchem.6c03044

**Published:** 2026-09-12

**Authors:** Raphael Neisius, Marco Moroni, Yali Yang, Sergio Marras, Matteo Lorenzoni, Paola Ragonese, Cecilia Daniela Costa, Mengjiao Wang, Giuseppe Ferraro, Giulia Folpini, Alessandro Stroppa, Lorenzo Malavasi, Teresa Gatti, Isabella Poli

**Affiliations:** † 121451 Istituto Italiano di Tecnologia, Center for Sustainable Future Technologies, Via Livorno 60, Torino 10144, Italy; ‡ 19032 Politecnico di Torino, Department of Applied Science and Technology, Corso Duca degli Abruzzi 24, Torino 10129, Italy; § Department of Chemistry and INSTM, 19001 University of Pavia, Via Taramelli 16, Pavia 27100, Italy; ∥ School of Mathematics and Physics, 12507 University of Science and Technology Beijing, Beijing 100083, China; ⊥ Istituto Italiano di Tecnologia, Materials Characterization Facility, Via Morego 30, Genova 16163, Italy; # Consiglio Nazionale Delle Ricerche, 96976 Institute for Photonics and Nanotechnology (CNR-IFN), Milano 20133, Italy; ∇ Istituto Italiano di Tecnologia, Center for Nano Science and Technology, Via Rubattino 81, Milan 20134, Italy; ○ Consiglio Nazionale delle Ricerche, Institute for Superconducting and Innovative Materials and Devices (CNR-SPIN), c/o Department of Physical and Chemical Sciences, University of L’Aquila, Coppito, L’Aquila I-67100, Italy

## Abstract

Lead-free low-dimensional metal halides are emerging as highly tunable materials for optoelectronic applications. Here, we report a new class of zero-dimensional bismuth halides incorporating phenethylammonium (PEA) cations, namely PEA_4_BiI_7_ and PEA_4_BiBr_7_, and investigate their structural, thermal, and optoelectronic properties. Single-crystal X-ray diffraction reveals that both compounds consist of isolated [BiX_6_] octahedra separated by bulky organic cations, although they crystallize in different crystal systems and exhibit high stability under ambient conditions. In contrast, the chloride analogue adopts a distinct stoichiometry, PEA_4_Bi_2_Cl_10_, featuring edge-sharing octahedral dimers, highlighting the strong impact of halide substitution on the structural connectivity. Optical measurements show a systematic bandgap evolution following the trend Cl > Br > I, with pronounced excitonic absorption features in all compounds; density functional theory calculations indicate that the conduction band edge is dominated by the inorganic Bi-X framework, while the valence band edge progressively changes from the organic cation to the inorganic sublattice. Thin films were fabricated via a simple one-step spin-coating procedure. Particularly, PEA_4_BiI_7_ forms highly oriented, phase-pure thin films and exhibits broadband visible photoluminescence with excitation-dependent behavior. These results establish phenethylammonium bismuth halides as a versatile platform for stable, lead-free zero-dimensional semiconductors with promising potential for solution-processed optoelectronic devices.

## Graphical abstract

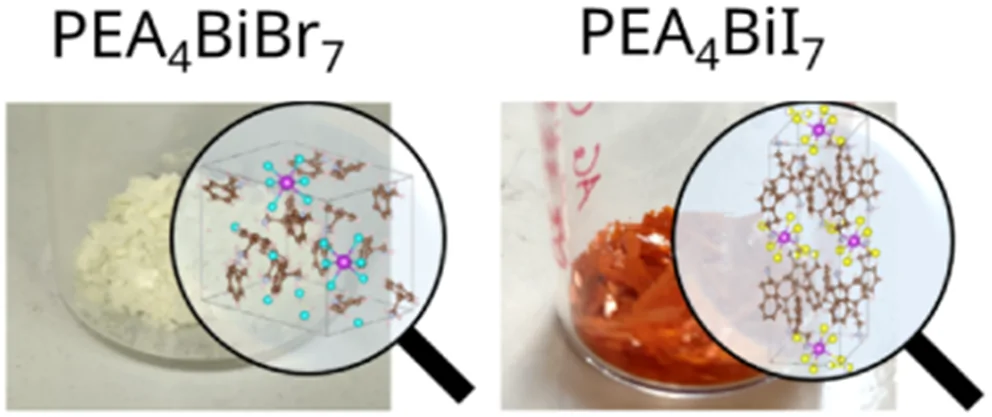

## Introduction

Three-dimensional lead-based perovskites have demonstrated remarkable performance in photovoltaic applications, achieving high efficiencies in both single-junction and tandem architectures and increasingly competing with established silicon technologies. Despite their outstanding performance, the presence of lead as the B-site cation raises environmental and health concerns.^1^ Significant efforts have been devoted to addressing these issues through strategies such as advanced encapsulation and in situ Pb sequestration to prevent leakage.^2,3^ Although recent studies indicate that lead released into aqueous environments can be rapidly immobilized in soil, a fraction may still remain bioavailable.^4^ These considerations have motivated the search for lead-free alternatives.^5^ Among these, tin-based perovskites have shown the greatest promise so far, with reported power conversion efficiencies exceeding 15% in single-junction devices.^6^ In parallel, bismuth-based halide materials have attracted increasing attention because of the similar electronic configuration of Bi^3+^ to Pb^2+^ and their potentially lower toxicity. One of the most studied bismuth-based systems is the dimeric compound with general formula A_3_Bi_2_I_9_, where A is either MA^+^= methylammonium or Cs^+^, characterized by face-sharing [BiI_6_]^3-^-octahedra. Cs_3_Bi_2_I_9_ has demonstrated power conversion efficiencies exceeding 1% in photovoltaic devices, along with enhanced stability toward moisture compared to three-dimensional lead-based perovskites.^7^ Although their photovoltaic performance remains limited, bismuth-based materials exhibit a wide range of functional properties, including X-ray detection,^8^ ferroelectricity,^9^ chromogenic behavior,^10–12^ and broadband luminescence.^13^

In this context, low-dimensional systems, particularly zero-dimensional (0D) structures, are especially attractive because of their strong carrier confinement and potential for highly efficient light emissions. In 0D structures, the inorganic building blocks are electronically isolated from one another by organic cations, suppressing long-range band dispersion and favoring exciton localization. Such confinement can promote efficient radiative recombination and the formation of self-trapped excitons, often resulting in broadband emission with large Stokes shifts and reduced self-absorption. These features make 0D halides promising candidates for light-emitting applications, phosphors, and radiation detection technologies. Moreover, the structural diversity accessible through organic-cation engineering offers broad opportunities to tune crystal packing, lattice distortion, and electron-phonon coupling, which are key parameters governing emission properties.^14^

While 0D tin-based materials have been extensively explored and exhibit high photoluminescence quantum yields (PLQY) in the red and yellow spectral regions,^15,16^ efficient emitters across the full visible range remain scarce. In particular, bismuth-based 0D systems remain comparatively underexplored despite the strong spin-orbit coupling and stereochemically active lone-pair electrons associated with Bi^3+^, both of which can strongly influence excited-state dynamics and emission behavior. Developing new bismuth-based low-dimensional halides is therefore of considerable interest for understanding structure–property relationships and for expanding the library of lead-free luminescent materials.

In this work, we report bismuth-based organic halides incorporating PEA cations, namely PEA_4_BiI_7_ and PEA_4_BiBr_7_, and investigate their structural and optoelectronic properties. A combination of single-crystal X-ray diffraction, thermal analysis, optical spectroscopy, and density functional theory (DFT) calculations is employed to elucidate the relationship between composition, structure, and electronic behavior. In addition, we explore the effect of halide substitution across the series, including the chloride analogue PEA_4_Bi_2_Cl_10_, and demonstrate the fabrication of thin films via a simple solution-based approach, enabling a direct assessment of their suitability for optoelectronic applications.

## Experimental Section

### Materials

Phenethylammonium bromide (PEABr), phenethylammonium iodide (PEAI, >98%), bismuth­(III) chloride (BiCl_3_, >98%), bismuth­(III) bromide (BiBr_3_, 99.998%), bismuth­(III) iodide (BiI_3_, 99%), hydrobromic acid (HBr, 48%), hexane (>99%), and acetonitrile (MeCN, >99.9%) were purchased from Sigma-Aldrich. Hydrochloric acid (HCl, 37%) and hydroiodic acid (HI, 55%) were purchased from Thermo Fisher Scientific. Phenethylammonium chloride (PEACl) was taken from own stocks. All chemicals were used without further purification.

### Synthesis

#### Preparation of PEACl Precursor

PEABr (3.186 g) was placed in a 50-mL-round bottle flask. After addition of 10 mL conc. HCl, the reaction mixture was heated to 120 °C in reflux conditions under continuous stirring, forming a yellowish transparent solution. After stirring for 1 h at 120 °C, the solution was cooled down to room temperature without stirring for 2 h and large colorless needles formed throughout the solution. After removing excess solvent, the crystals are washed three times with 20 mL of hexane. The colorless crystals are dried overnight under vacuum at 60 °C. The yield based on the Br precursor was 95.5%.

#### Preparation of Single Crystals

Single crystals were grown employing a hydrothermal synthesis approach that had been employed in earlier works to grow two-dimensional bismuth-based double perovskites.^14^

*PEA*_*4*_ *Bi*_*2*_ *Cl*_*10*_. 1.5 g of a 4:1 molar mixture of PEACl (1000.35 mg) and BiCl_3_ (500.59 mg) was dissolved in 10 mL of conc. HCl. The reaction mixture was heated up to 120 °C under continuous stirring forming a transparent mild-yellowish solution. After stirring for 1h, the solution cooled back to room temperature over 2 h, precipitating a colorless solid. After removing the excess solvent by decantation, the colorless crystals are washed three times with 20 mL of hexane and dried overnight in a vacuum oven at 60 °C. The yield based on the Bi precursor was 98.2%.

*PEA*_*4*_ *BiBr*_*7*_. 2 g of a 4:1 molar mixture of PEABr (1286.39 mg) and BiBr_3_ (714.62 mg) was dissolved in 10 mL of conc. HBr. The reaction mixture is heated up to 120 °C under continuous stirring, forming a clear yellowish solution. After stirring for 2 h, the solution was allowed to cool back to room temperature for 2 h precipitating a faint-yellow solid. After removing the excess solvent by decantation, the colorless crystals are washed three times with 20 mL of hexane and dried overnight in a vacuum oven at 100 °C. The yield based on the PEA precursor was 63.1%.

*PEA*_*4*_ *BiI*_*7*_. 2 g of a 4:1 molar mixture of PEAI (1256.62 mg) and BiI_3_ (743.92 mg) was dissolved in 10 mL of conc. HI. The reaction mixture is heated up to 120 °C under continuous stirring, forming a dark-red solution. After stirring for 2 h, the solution was allowed to cool back to room temperature overnight, thereby precipitating a dark-red solid. After removing the excess solvent by decantation, the red-orange crystals are washed three times with 20 mL of hexane and dried overnight in a vacuum oven at 100 °C. The yield based on the PEA precursor was 76.8%.

#### Deposition of Thin Films

0.15 mmol of PEA_4_Bi_2_Cl_10_ and PEA_4_BiI_7_ was added to 1 mL of MeCN and stirred at 40 °C till clear colorless and red solutions are obtained. 0.125 mmol of PEA_4_BiBr_7_ was added to 1 mL of MeCN and stirred at 60 °C till a clear yellow solution is formed. Then, 100 μL of these transparent solutions was spin coated onto a 25 mm × 25 mm glass substrate or an FTO coated substrate at 1500 rpm for 30 s with a ramp of 500 rpm s^–1^. The thin films were subsequently annealed for 10 minutes at 60 °C.

### Characterization

Single-crystal X-ray diffraction (SCXRD) measurements on selected crystals were collected on a Rigaku XtaLab SuperNova (Rigaku Europe SE, Neu-Isenburg, Germany) four-circle diffractometer equipped by a microfocus X-ray source (λ = 0.7107 Å) and a Dectris Pilatus 200K hybrid pixel array detector (DECTRIS AG, Baden-Daettwil, Switzerland). Data collection, data reduction, and accurate measurements of the unit cell parameters were performed by means of the CrysAlisPro software suite, ver. 1.171.41 (Rigaku Oxford Diffraction 2021; CrysAlisPro Software system, version 1.171.41.104a, Rigaku Corporation, Wroclaw, Poland). Absorption effects were empirically evaluated based on a multiscan approach using spherical harmonics by the SCALE3 ABSPACK scaling algorithm implemented in the CrysAlisPro crystallographic suite. Crystal structures were solved by direct methods (SIR 97) and refined by full-matrix least-square procedures on F2 using all reflections (SHELXL 2018/3).^17,18^ The crystal structures have been deposited in the Cambridge Crystallographic Data Centre (CCDC codes 2584841 and 2584842), and the corresponding CIF files are included in the Supporting Information.

X-ray diffraction (XRD) analysis of thin films was carried out on a Malvern-PANalytical 3rd generation Empyrean X-ray powder diffractometer. The instrument was equipped with a 1.8 kW CuKα X-ray tube operating at 45 kV and 40 mA, iCore and dCore automated PreFIX optical modules, a motorized Eulerian Cradle (chi, phi,x,y and z movements), and a solid-state hybrid pixel PIXcel3D detector acquiring in 1D mode. The XRD patterns were collected in Bragg-Brentano geometry over a 2θ range of 5–70°, with a step size of 0.026°. Pole figure measurements were performed in parallel-beam (PB) geometry using an X-ray lens, a motorized Eulerian cradle, a 0.28° parallel plate collimator (PPC), and a PIXcel3D hybrid pixel detector operating in 0D mode. Data were collected with a step size of 5° in φ and 4° in χ, covering χ angles from 0° to 88°. Measurements were carried out in air at room temperature, using a zero-diffraction silicon substrate. Pole figure data were processed using X’Pert Texture (version 1.3, Malvern Panalytical), while the simulated XRD pattern was generated using CrystalDiffract (version 7.2.4, CrystalMaker®).

Powder X-ray diffraction (PXRD) measurements were performed with a PANalytical Empyrean Diffractometer with Cu-α radiation source (λ = 1.54184 Å) operating at 40 kV and 40 mA in the 2θ within 5° to 70°, employing a step size 0.0131° and 349.095 s per step. PXRD data were compared to calculated structural data from SCXRD using the Rietveld refinement method, employing PROFEX software for modeling.^19^

Reflectance spectra of pellets and absorption spectra of thin films were collected for the wavelength range from 200 nm to 800 nm using a photospectrometer PerkinElmer Lambda 650S.

Thin-film and single-crystal photoluminescence (PL) at room temperature was measured in a NanoLog spectrofluorometer (Horiba Jobin Yvon), selecting excitation wavelengths of 480 nm (I-based material), 380 nm (Br and I), and 340 nm (Cl). The temperature- and pump-fluence-dependent photoluminescence measurements were conducted under fs laser excitation conditions, using the output of a Yb laser (Light Conversion Pharos, 1030 nm wavelength, 500 kHz repetition rate) coupled to an OPA system (Orpheus) set either at 480 nm or 380 nm, using the OPA signal output converted to its 2nd harmonic (Lyra). The OPA output was focused on the sample using a 30 cm focal lens to a 50 μm FWHM spot; the samples’ PL was then measured with a visible spectrometer (Ocean Optics Maya). For these measurements, the thin-film samples were kept under vacuum conditions inside a Linkam cryostat, which was used to control the sample temperature between 77 K and 430 K.

Thermogravimetric analyses (TGA) were conducted with a Netzsch TG209 F1 “Libra” up to 700 °C at a heating rate of 10 °C/min in air and N_2_ flux.

Differential scanning calorimetry (DSC) measurements were carried out using a Netzsch DSC 204 F1 “Phoenix” calorimeter in the temperature range of −70 °C to 150 °C at a heating and cooling rate of 20 °C min^–1^ under N_2_ flux. Each sample was exposed to three heating and cooling cycles, and the glass transition temperature was determined from the inflection point of the glass transition.

Atomic force microscopy (AFM) topography measurements were performed in noncontact (NC) mode using a Park Systems XE-100 atomic force microscope under ambient conditions. Silicon cantilevers (OMCL-AC160TS, Olympus/Bruker compatible) with a nominal resonance frequency of 300 kHz were used for imaging. The acquired topographic images were analyzed and processed by using the Gwyddion software package, including standard background leveling and flattening procedures.

Field emission scanning electron microscopy (FESEM) images were obtained using a ZEISS SUPRA 40 field-emission scanning electron microscope equipped with an InLens detector. Top-view images were acquired at an accelerating voltage of 3 kV using a 30 μm aperture on the thin films deposited on FTO substrates.

#### Computational Details

All density functional theory (DFT) calculations were performed with the VASP package.^20^ The generalized gradient approximation (GGA), in the form of the Perdew–Burke–Ernzerhof (PBE) functional, was employed to describe the exchange-correlation interactions, while the projector augmented wave (PAW) method was used to treat the core electrons.^21,22^ The plane-wave cutoff was set to 520 eV, and the convergence criteria for energy and force were set to 10^–6^ eV and 0.01 eV/Å, respectively. The PBE-D3 method with Becke-Johnson damping was used to describe the van der Waals interactions.^23^ During structural optimization, atomic positions were fully relaxed, while the lattice parameters were fixed to their experimental values. The k-mesh is set to 3 × 1 × 1, 2 × 2 × 2, and 3 × 4 × 1 for (PEA)_4_Bi_2_Cl_10_, (PEA)_4_BiBr_7_, and (PEA)_4_BiI_7_, respectively, when optimizing the structures. The high-symmetry k-points in the irreducible Brillouin zone were obtained through VASPKIT package for subsequent band structure calculations.^24^

## Results and Discussion

Phenethylammonium bismuth halide single crystals were synthesized hydrothermally by mixing phenethylammonium and bismuth halide precursors in a 4:1 molar ratio to target the PEA_4_BiX_7_ composition, with further details being provided in the Experimental Section.

Crystal structures were determined using SCXRD, revealing chemical formulas of PEA_4_BiI_7_, PEA_4_BiBr_7_ and PEA_4_Bi_2_Cl_10_ as shown by the crystallographic data summarized in Table 1. Refined atomic coordinates are shown in the Supporting information. The crystal structure of PEA_4_Bi_2_Cl_10_ has already been reported and made available.^25^ PEA_4_BiBr_7_ has been reported so far only in its monohydrate form.^26^ To our best knowledge, the crystal structures of water-free PEA_4_BiBr_7_ and PEA_4_BiI_7_ have not been reported before. Figures 1 a, b and Figure S1 show the three-dimensional crystal structures of the bismuth halide compounds as determined by SCXRD results which show a 0D structure, while the calculated XRD patterns are shown in Figure S2 with Miller indices assigned to all dominant reflexes in the 2θ range from 5° to 25° (Figure S3). PEA_4_Bi_2_Cl_10_ crystallizes in a monoclinic crystal system of the P2_1_/c space group in which isolated [Bi_2_Cl_10_]^4–^ dimers, consisting of two edge-sharing octahedra, are surrounded by organic PEA cations (Figure S1), as already reported in the literature.^25^ PEA_4_BiBr_7_ instead forms triclinic crystals of the P1 space group, which consists of isolated [BiBr_6_]^3–^-octahedra separated by layers of PEA cations, as presented in Figure 1a. Despite not containing crystal water, PEA_4_BiBr_7_ maintains a similar crystal structure close to the previously reported monohydrate.^26^ PEA_4_BiI_7_, which has not been reported elsewhere, features similar crystallographic features to the bromide compound, with layers of isolated [BiI_6_]^3–^ octahedra and single I^–^ ions separated by layers of PEA cations, while assembling in a monoclinic crystal of the P2_1_/c space group, like the chloride compound.

**Table 1:** Crystallographic Data of (PEA)_4_Bi_2_Cl_10_, (PEA)_4_BiBr_7_, and (PEA)_4_BiI_7_

Empirical formula	(C_8_H_12_N)_4_Bi_2_Cl_10_	(C_8_H_12_N)_4_BiBr_7_	(C_8_H_12_N)_4_BiI_7_
Formula Weight	1261.2	1257.09	1586.02
Temperature	298(2) K	298(2) K	298(2) K
Wavelength	0.7107 Å	0.7107 Å	0.7107 Å
Crystal system	Monoclinic	Triclinic	Monoclinic
Space Group	P2_1_/c	P1	P2_1_/c
Unit cell dimensions	a = 8.0742(0) Å	a = 13.2450(4) Å	a = 10.7110(6) Å
	b = 25.3398(0) Å	b = 13.2880(5) Å	b = 8.5739(3) Å
	c =21.9976(0) Å	c = 13.4205(6) Å	c = 26.1410(12) Å
	α = 90.000(0)°	α = 102.518(3)°	α = 90.000(0)°
	β = 90.592(0)°	β = 93.427(3)°	β = 100.958(4)°
	γ = 90.000(0)°	γ = 109.470(3)°	γ = 90.000(0)°
Volume, *Z*	4500.4381 Å^3^, 4	2151.51(15) Å^3^, 1	2356.89(19) Å^3^, 2
Density (calculated)	1.816 mg m^–3^	1.940 mg m^–3^	2.235 mg m-3
Final *R* indices	*R*_1_ = 0.0606	*R*_1_= 0.0849	*R*_1_= 0.0670
	w*R*_2_ =0.1843	w*R*_2_ =0.2460	w*R*_2_ =0.1728
*R* indices (all data)	*R*_1_ = 0.0956	*R*_1_ = 0.1171	*R*_1_= 0.1343
	w*R*_2_ =0.2045	w*R*_2_ = 0.2713	w*R*_2_ =0.2098

**Figure 1:**
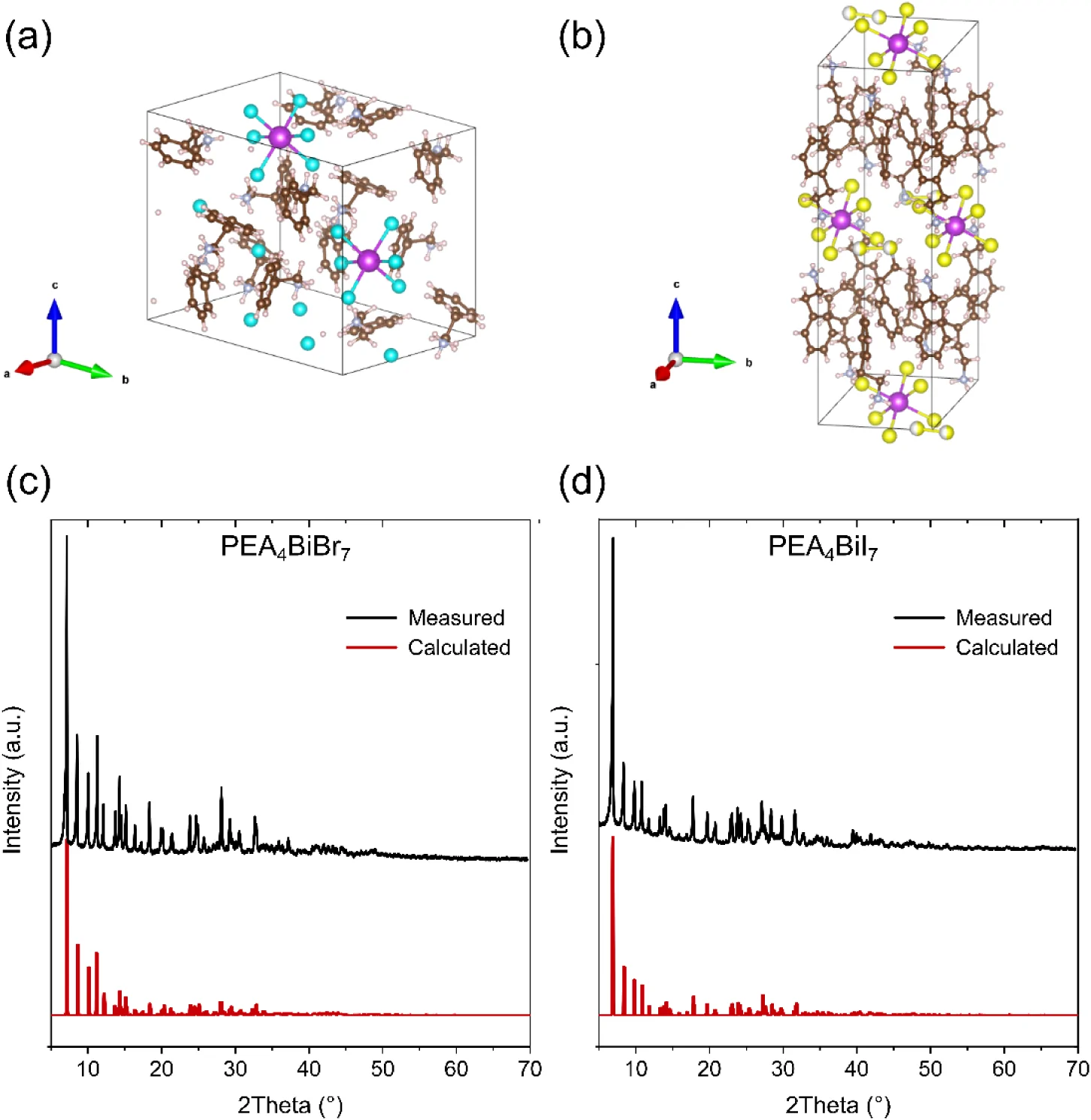
Three-dimensional crystal structures of (a) PEA_4_BiBr_7_ and (b) PEA_4_BiI_7_ determined by the single-crystal X-ray diffraction results. Magenta: Bi, cyan: Br, yellow: I, brown: C, pink: H, and violet: N. Calculated XRD patterns from single-crystal characterization and powder XRD patterns of (c) PEA_4_BiBr_7_ and (d) PEA_4_BiI_7_.

Figure 1b depicts the crystal structure of the novel iodide compound. In contrast to divalent metal halide systems, such as Sn^2+^ and Pb^2+^, where charge neutrality is achieved with isolated ${\left[MX_{6}\right]}^{4-}$ octahedra, trivalent Bi^3+^ leads to ${\left[BiX_{6}\right]}^{3-}$ units; charge compensation is achieved through the incorporation of an extra halide anion that remains isolated and only interacts with the protonated amino groups, resulting in a PEA_4_BiX_7_ stoichiometry. The calculated results were experimentally verified by comparing them with powder diffractograms of ground single crystals. The XRD patterns shown in Figures 1c–d and Figure S4 indicate good consistency with the calculated SCXRD patterns. The phase purity of the bulk samples was confirmed by Rietveld refinement of the PXRD data using PROFEX, employing the corresponding single-crystal structures as the initial structural models. The refinement plots and crystallographic parameters are provided in the Supporting Information (Figures S5–S7 and Table S1).

Thermal and phase stability of the newly reported materials was monitored by thermogravimetric analysis (TGA) in both N_2_ and air and differential scanning calorimetry (DSC) analysis. TGA measurements shown in Figure S8 indicate that PEA_4_BiBr_7_ and PEA_4_BiI_7_ are thermally stable up to 200 °C under N_2_. Above this temperature, the thermal degradation of PEA halides begins, leading to the formation of phenethylamine and the corresponding hydrogen halides.^27^ Further heating causes the material components, particularly PEA^+^, to melt and thermally decompose.^27–29^ As shown in Figure S9, the TGA measurements performed in air display a similar overall trend, although slight differences are observed due to oxidation processes occurring under oxidative atmosphere. While the phenethylammonium halides decompose similarly up to approximately 400 °C, the bismuth trihalides undergo a sequence of oxidation reactions, forming intermediate bismuth oxyhalides before ultimately converting into Bi_2_O_3_.^30,31^ DSC measurements covering a temperature range from −70 °C to 150 °C display no sharp melting peaks, reaffirming that melting occurs at temperatures >150 °C (Figure S10), which is in line with the TGA results and the reported melting points of the precursor materials, which are all above 200 °C.^28,29^ No first-order phase transitions are observed, and all materials show a step in heat flow (Δ*C*_p_) with an overall reversible behavior, which indicates a glass transition (*T*_g_) of the organic-inorganic lattice at 47.8 °C for PEA_4_BiBr_7_ and 129.5 °C for PEA_4_BiI_7_.

The DSC results can be directly correlated with the crystallographic differences between the two compounds. PEA_4_BiI_7_, which exhibits a high *T*_g_ of 129.5 °C, crystallizes in the higher-symmetry monoclinic *P2*_*1*_ */c* space group and shows a significantly higher calculated density, indicating a more efficiently packed and ordered lattice with reduced free volume. The presence of two formula units per unit cell (Z = 2) and the anisotropic unit cell, featuring a substantial difference between the values of the three axes, further suggest a more cooperative and constrained structural arrangement. In contrast, PEA_4_BiBr_7_, with a much lower *T*_g_ of 47.8 °C, adopts a triclinic *P1* structure with lower symmetry, lower density, and greater structural freedom, consistent with increased disorder and higher free volume. Together, these features indicate that the iodide analogue forms a more rigid and tightly packed lattice with stronger overall intermolecular interactions, which suppress molecular mobility and result in a higher glass transition temperature compared with the more flexible bromide analogue.

Starting from single crystals, finely ground powders were prepared and subsequently used to fabricate thin films via spin coating. The powders were dissolved in acetonitrile to form stable inks, which were deposited onto glass substrates to obtain the films. The UV-Vis spectra of the precursor inks (Figure S11) retain the characteristic absorption onset of the corresponding [BiX_6_]^3–^ anions in acetonitrile, indicating that the material sufficiently dissolves in the chosen solvent.^32–34^ Figure S12 shows pictures of the obtained thin films, while Figure 2 a, b, and c shows the surface morphology of PEA_4_Bi_2_Cl_10_, PEA_4_BiBr_7_ and PEA_4_BiI_7_, respectively. PEA_4_BiBr_7_ and PEA_4_BiI_7_ exhibit the formation of flower-like morphologies, typical of bismuth halide compounds and low-dimensional perovskites.^35,36^ The iodide-based compound shows branched growth, with larger flowers than the bromide material, suggesting a more controlled growth. The PEA_4_Bi_2_Cl_10_ film instead exhibits a heterogeneous surface morphology characterized by extended crack-like features and regions of compact homogeneous coverage composed of densely packed rounded grains. Figure S13 shows the AFM topographical profiles used to determine the thin-film thicknesses. PEA_4_BiBr_7_ and PEA_4_BiI_7_ films exhibit an average thickness of 1.47±0.34 and 1.08±0.04 μm, respectively, whereas PEA_4_Bi_2_Cl_10_ films are considerably thinner, with an average thickness of 0.64±0.02 μm, and display a more homogeneous morphology overall. This behavior is consistent with the more compact surface coverage observed for the chloride compound compared to the flower-like morphology exhibited by the bromide- and iodide-based films. To quantitatively evaluate the surface morphology, AFM measurements were performed over 20 × 20 μm areas for all films (Figure S14). The extracted RMS roughness values are approximately 36 nm, 62 nm, and 110 nm for PEA_4_Bi_2_Cl_10_, PEA_4_BiBr_7_, and PEA_4_BiI_7_, respectively. In addition, the surface coverage, estimated from the AFM topographic images, was found to be approximately 95%, 90%, and 75% for the chloride, bromide, and iodide films, respectively. These results quantitatively confirm the trends observed in the SEM images: PEA_4_Bi_2_Cl_10_ forms the most continuous film with the highest substrate coverage, whereas PEA_4_BiBr_7_ and PEA_4_BiI_7_ exhibit a rougher and less continuous morphology.

**Figure 2:**
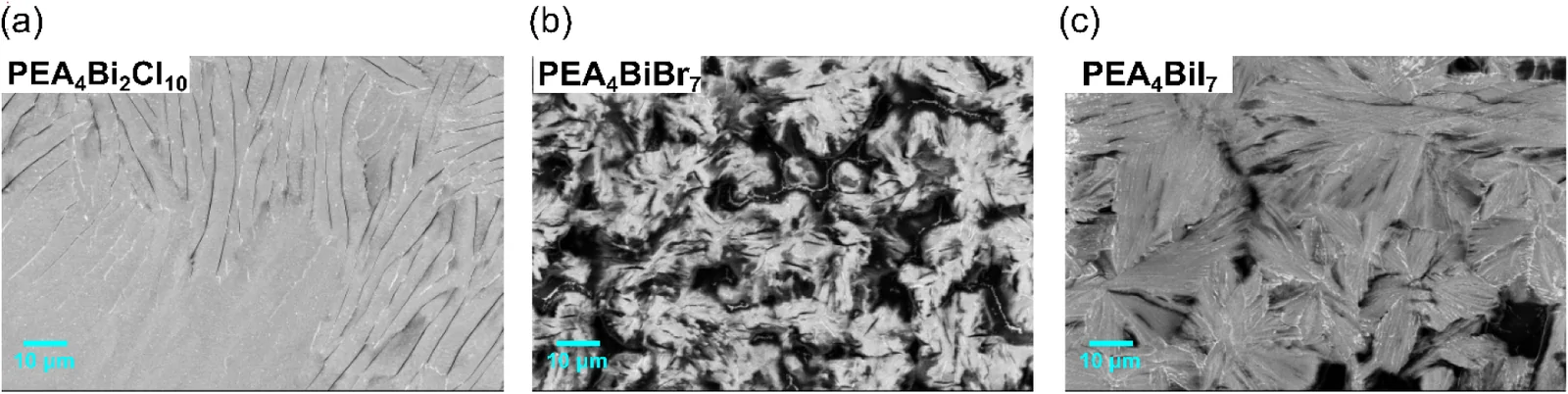
Top-view SEM images of (a) PEA_4_Bi_2_Cl_10_, (b) PEA_4_BiBr_7_, and (c) PEA_4_BiI_7_.

The thin-film XRD patterns shown in Figure 3 a, b, and c reveals a clear evolution in crystallographic orientation and phase purity across the halide series. PEA_4_BiI_7_ shows a highly intense diffraction pattern dominated by a series of (00l) reflections, indicating a strong preferred orientation along the c-axis. The absence of additional peaks and the high intensity suggest that the film is monophasic and highly textured, with crystallites well aligned perpendicular to the substrate. In contrast, PEA_4_BiBr_7_ exhibits peaks corresponding to the (h00) reflection, consistent with its single-crystal structure, but also shows an additional intense peak at 6.8°, which indicates the formation of a secondary phase with larger spacing. Importantly none of the starting precursors (PEABr and BiBr_3_)^37,38^ exhibit reflections at this angle, ruling out their contribution to this feature. We believe the secondary phase might be related to binary Bi_2_Br_10_-containing species, which have shown to produce reflections at such low diffraction angles.^39^ The reduced overall intensity compared to the iodide analogue indicates multiphase crystallization and less controlled film growth (Figure S15). A similar behavior is observed for PEA_4_Bi_2_Cl_10_, where the overall diffraction intensity is significantly lower and the amorphous background from the glass substrate is more pronounced. While a preferential orientation along the (0k0) direction is visible, additional weak reflections are also present, as indicated by the asterisks, suggesting minor impurity phases or structural heterogeneity. Besides differences in crystallinity, the markedly reduced intensity in the chloride-based material may also arise from the substantially lower thickness. The specific preferred orientation of each material is shown in Figure 3d, e, and f.

**Figure 3:**
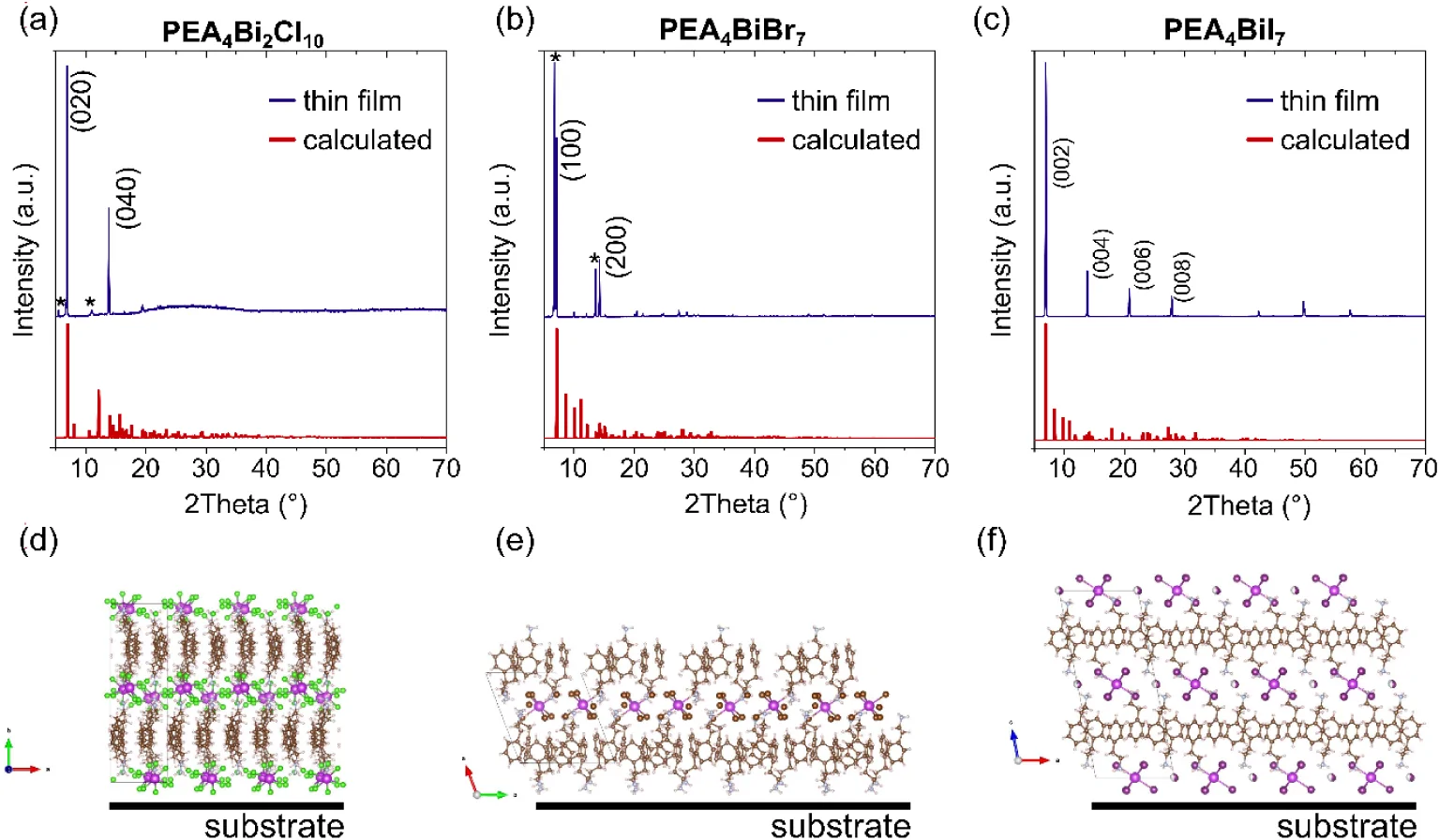
XRD patterns of (a) PEA_4_Bi_2_Cl_10_, (b) PEA_4_BiBr_7_ and (c) PEA_4_BiI_7_ thin films. The asterisks in panels a and b denote peaks that do not belong to the primary phase. Schematic diagrams illustrating the orientation of (d) PEA_4_Bi_2_Cl_10_, (e) PEA_4_BiBr_7_, and (f) PEA_4_BiI_7_ thin films.

The experimental θ–2θ diffraction pattern of PEA_4_BiI_7_ was compared with a diffraction pattern simulated from the reported crystal structure using CrystalDiffract v7.2.5, assuming a fully (100%) c-axis-oriented crystallite distribution representative of an ideally textured film. As shown in Figure S16, an excellent agreement is observed between the experimental and simulated diffraction profiles, confirming that the observed reflections are those expected for a highly c-axis-oriented film. Within the detection limits of the measurement, no additional reflections arising from differently oriented crystallites or secondary phases are observed, indicating a strong preferential orientation with the crystallographic c-axis perpendicular to the substrate.

To quantitatively assess out-of-plane texture, X-ray texture analysis was performed by measuring the Pole Figure (PF) of the (002) reflection. As shown in Figure 4a, the PF exhibits a single intense maximum centered at *χ* = 0°, indicating that the (002) planes are preferentially aligned parallel to the substrate surface. The corresponding *χ* profile yields a full width at half maximum (FWHM) of 18.1° (Figure 4b) and the top-view PF displays an almost circular intensity distribution (Figure S17), indicating a limited angular distribution of crystallite orientations and a well-defined out-of-plane texture (Figure 4b). These complementary analyses provide quantitative evidence for the pronounced preferred orientation inferred from the θ–2θ XRD pattern and demonstrate that the PEA_4_BiI_7_ thin films possess a well-defined out-of-plane crystallographic texture.

**Figure 4:**
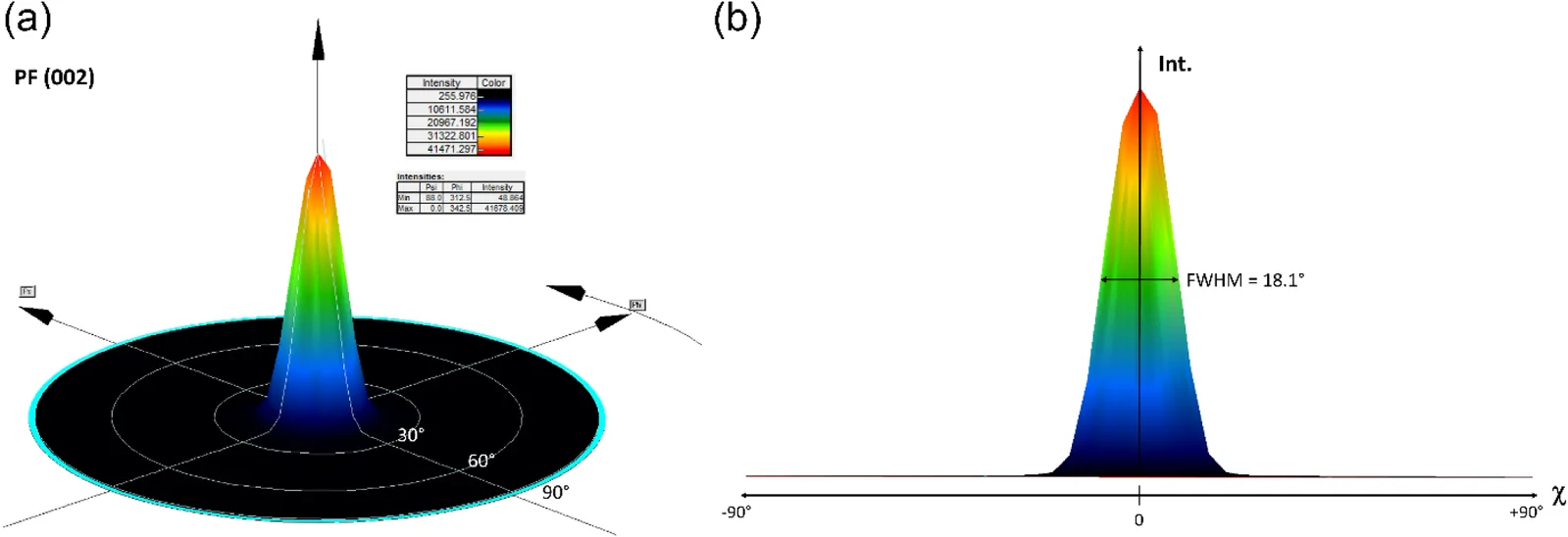
Pole figure analysis of a PEA_4_BiI_7_ thin film based on the (002) reflection; (a) three-dimensional tilted view of the pole figure; (b) side view (*χ*-profile) showing the angular spread of the out-of-plane texture, with a full width at half maximum (FWHM) of 18.1°.

The optical properties of the three compounds were studied using UV-Vis spectroscopy. First, finely ground powders were compressed into ultrasmooth pellets using a hydraulic press. These pellets were measured in diffuse reflectance mode and compared with thin films analyzed in transmission mode. Figure S18 shows the diffuse reflectance spectra of the pellets. Assuming an indirect bandgap for these semiconductor materials,^40–42^ the optical gaps were estimated using the Kubelka-Munk function, yielding values of 2.93, 2.73, and 2.11 eV for PEA_4_Bi_2_Cl_10_, PEA_4_BiBr_7_, and PEA_4_BiI_7_, respectively (Figure 5a). Figure 5b shows the UV-Vis absorption spectra of the corresponding thin films measured in transmittance mode. PEA_4_BiI_7_ and PEA_4_BiBr_7_ exhibit pronounced absorption features at approximately 480 nm and 380 nm, respectively, which can be attributed to the excitonic transitions, followed by a second absorption onset. PEA_4_Bi_2_Cl_10_ shows an excitonic peak around 350 nm; however, because it lies in the deep-UV region, it is not possible to clearly resolve the presence of a second absorption onset. To distinguish the excitonic contribution from the continuum-state absorption, the absorption spectra near the band edge were fitted using the Elliott model (see the Supporting Information for the fitting procedure and corresponding equations).^43,44^ The complete fitting results are reported in Figure S19 and Table S2, while the extracted bandgap energy *E*_g_, excitonic transition energy *E*_ex_, and exciton binding energy *E*_b_ are summarized in Table 2. For PEA_4_Bi_2_Cl_10_, only the excitonic contribution could be fitted because the continuum absorption lies beyond the accessible spectral range. The values obtained from the Elliott model are in good agreement with those estimated by using the first-derivative analysis and reflectance measurements. Specifically, for PEA_4_BiI_7_, the first-derivative method yields a fundamental bandgap of 2.83 eV (Figure S20a), while the lower-energy feature at 2.46 eV corresponds to the excitonic transition. For PEA_4_BiBr_7_, the reflectance measurements assuming an indirect band gap result in a value of approximately 3.5 eV (Figure S20b). The bandgaps estimated from thin films are larger than those obtained from pellet measurements. This discrepancy can be attributed to the presence of strong excitonic effects in the thin films; in compressed powders, the excitonic features are broadened or partially suppressed due to scattering and structural disorder, leading to an apparent narrowing of the optical gap.

**Figure 5:**
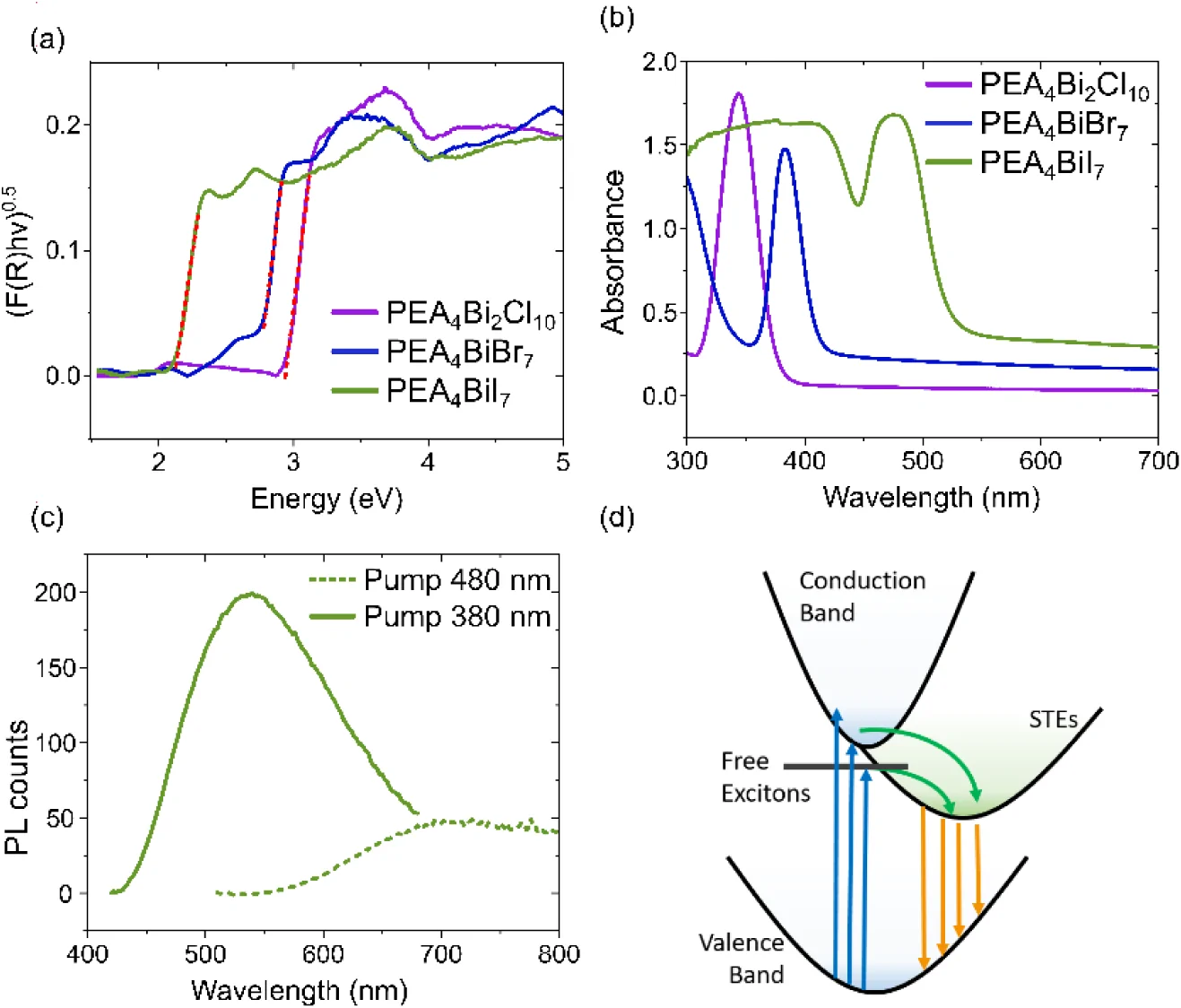
(a) Energy bandgaps of PEA_4_Bi_2_Cl_10_, PEA_4_BiBr_7_ and PEA_4_BiI_7_ powder pellets using the reflection-mode UV-Vis measurement. The red dotted lines represent the fit to experimental data. (b) UV-Vis absorption of PEA_4_Bi_2_Cl_10_, PEA_4_BiBr_7_, and PEA_4_BiI_7_ thin films using the transmission mode. (c) PL spectra of PEA_4_BiI_7_ thin film excited at 380 nm (solid line) and 480 nm (dotted line). (d) Illustration of the evolution of self-trapped exciton emission of PEA_4_BiI_7_.

**Table 2:** Energy Values of *E*_g_, *E*_ex_, and *E*_b_ Obtained from Fitting the Near Band Edge Absorption with the Elliott Model

Compound	*E*_g_ (eV)	*E*_ex_ (eV)	*E*_b_ (eV)
PEA_4_Bi_2_Cl_10_	-	3.60	-
PEA_4_BiBr_7_	3.87	3.23	0.65
PEA_4_BiI_7_	2.86	2.60	0.26

Figure S21 shows the photoluminescence (PL) peaks of the bismuth halide thin films. All films were excited at photon energies resonant with their excitonic absorption peaks, namely 340, 380, and 480 nm for PEA_4_Bi_2_Cl_10_, PEA_4_BiBr_7_, and PEA_4_BiI_7_, respectively. Upon resonant excitation, all compounds exhibit broad PL bands extending over the visible range with emissions characterized by pronounced Stokes shifts. In particular, PEA_4_Bi_2_Cl_10_ and PEA_4_BiBr_7_ exhibit asymmetric emissions centered around 550 nm, with a long tail extending toward lower energies, indicating a distribution of emissive states rather than a single recombination channel. PEA_4_BiI_7_ instead exhibits a broad and weak emission band at lower energies, centered around 725 nm. The observed broad and red-shifted emission across all compositions is consistent with recombination mediated by self-trapped excitons (STEs). In 0D metal halide systems, the strong spatial confinement of carriers within isolated octahedra enhances electron-phonon coupling, promoting lattice distortion upon excitation and stabilizing localized excitonic states.^45–48^ This results in large Stokes shifts and broadband emission. The asymmetry of the PL peak further supports the presence of multiple emissive configurations, likely associated with a distribution of lattice distortions or trapping sites (Figure 5d).

To further verify the excitonic nature of the emission, we performed excitation fluence-dependent PL measurements of PEA_4_BiI_7_ (Figure S22). The absence of saturation over the investigated fluence range and the nearly linear scaling of the PL intensity are characteristic of excitonic recombination and rule out a dominant contribution from defect-related luminescence, providing further evidence that the broadband emission originates from multiple self-trapped exciton states. Furthermore, temperature-dependent PL measurements performed under 380 nm excitation (Figure S23) provide additional support for this assignment. While the spectral shape remains essentially unchanged over the entire temperature range, the integrated PL intensity exhibits a non-monotonic behavior. As expected, the PL intensity initially decreases from 77 to approximately 150 K due to enhanced non-radiative scattering. Above ∼160 K, however, the PL intensity progressively increases up to about 390 K, consistent with the thermal activation of trapped carriers and their release into radiative self-trapped exciton states. This behavior indicates that defects primarily act as nonradiative recombination centers rather than emissive centers. Above 390 K, the PL intensity decreases again, coinciding with the glass phase transition occurring at approximately 401 K, as confirmed by DSC measurements.

Importantly, when the iodide-based film is excited at 380 nm, corresponding to excitation in the free carrier region, a more intense and relatively narrower emission centered at 550 nm appears, similar to that observed in the chloride and bromide analogues (Figure 5c). When combined with excitation fluence and temperature-dependent measurements, this behavior suggests the activation of alternative radiative pathways, likely involving shallow self-trapped excitons. Overall, the coexistence of these channels and dependence on excitation energy indicate a complex excited-state landscape governed by strong electron–phonon coupling.

Finally, we have investigated the electronic structures of the synthesized bismuth halide compounds on the DFT level using the Perdew–Burke–Ernzerhof (PBE) functional as implemented in the Vienna ab initio simulation package (VASP), initially without including SOC.^20^ The DFT input models were generated by selecting one ordered arrangement for each partially occupied motif and then relaxing the atomic coordinates while keeping the experimental lattice parameters fixed. Figure S24 shows the local inorganic motifs in the ordered relaxed models of PEA_4_Bi_2_Cl_10_, with edge-sharing [Bi_2_Cl_10_]^4–^ bioctahedra and PEA_4_BiX_7_ (X = Br, I), with isolated [BiX_6_]^3–^ octahedra charge-balanced by an additional halide anion. The ordered relaxed structures preserve the expected halide-size trend (Table 3). The mean Bi–X distances increase from 2.76 Å in PEA_4_Bi_2_Cl_10_ to 2.89 Å in the bromide and 3.11 Å in the iodide. In PEA_4_Bi_2_Cl_10_, two chloride ligands bridge adjacent Bi centers, and the intradimer Bi···Bi separation decreases slightly upon relaxation, from 4.393 Å in the experimental structure to 4.309 Å in the ordered model. In the bromide and iodide, by contrast, the additional halide remains well outside the first coordination sphere (> 6 Å from Bi), confirming that the relevant inorganic unit for the electronic-structure discussion is an isolated [BiX_6_] octahedron rather than an extended Bi–X–Bi framework.

**Table 3:** Selected Local Structural Metrics from the Experimental and Ordered Relaxed Structures^a^

Compound	Inorganic motif	Experimental Bi-X range (Å)	Relaxed Bi-X range (Å)	Additional note
PEA_4_Bi_2_Cl_10_	Edge-sharing [Bi_2_Cl_10_]^4–^ dimer	2.57–3.06	2.56–3.01	Two bridging Cl atoms; Bi···Bi = 4.39 Å (experimental) – 4.31 Å (relaxed).
PEA_4_BiBr_7_	Isolated [BiBr_6_]^3–^ octahedra + extra Br site	2.79–2.95	2.83–2.97	Additional Br remains > 6.1 Å from Bi.
PEA_4_BiI_7_	Isolated [BiI_6_]^3–^ octahedra + extra I site	3.05–3.06	3.08–3.16	Additional I remains > 6 Å from Bi; ordered model slightly broadens the Bi-I distribution.

a The bromide contains two crystallographically independent Bi centers and was refined in P1, whereas the chloride and iodide were monoclinic P21/c. In all ordered models, the experimental cell metrics were retained, and only the disordered motifs were resolved before coordinate relaxation.

The calculated band structures in Figure S25 display very flat bands near the band edges, consistent with weak electronic coupling among the low-connectivity Bi-halide units separated by bulky PEA cations. Within the calculations performed without SOC, the band gap decreases from 3.42 eV for PEA_4_Bi_2_Cl_10_ to 3.40 eV for PEA_4_BiBr_7_ and 2.59 eV for (PEA)_4_BiI_7_, reproducing the experimental ordering Cl > Br > I (Table 3). The Cl-to-Br difference is modest, whereas the iodide shows the expected red shift associated with the heavier halide. The projected densities of states in Figure S26 indicate that the conduction-band edge is dominated by the inorganic Bi-X sublattice in all three compounds. The valence-band edge evolves more strongly across the series: PEA_4_Bi_2_Cl_10_ retains a larger organic contribution, PEA_4_BiBr_7_ is more mixed, and PEA_4_BiI_7_ becomes clearly more inorganic. This progressive transfer of valence-edge weight from the organic cation to the inorganic sublattice provides a chemically intuitive rationale for the observed gap narrowing from chloride to iodide. The absolute agreement is not uniform across the series, however, so the calculations are most robust here for relative trends in band dispersion and orbital character rather than for quantitative prediction of the gap magnitude. For PEA_4_BiBr_7_ and PEA_4_BiI_7_, explicit inclusion of spin–orbit coupling reduces the calculated gap from 3.40 and 2.59 eV to 2.67 and 1.86 eV, respectively, showing that relativistic effects substantially renormalize the frontier electronic structure (Figure 6). This large shift is consistent with the predominant Bi-halide character of the conduction-band edge. At the same time, the SOC-inclusive value does not improve agreement with experiment within the present computational setup, which means that SOC is essential but not by itself sufficient for quantitative gap prediction.

**Figure 6:**
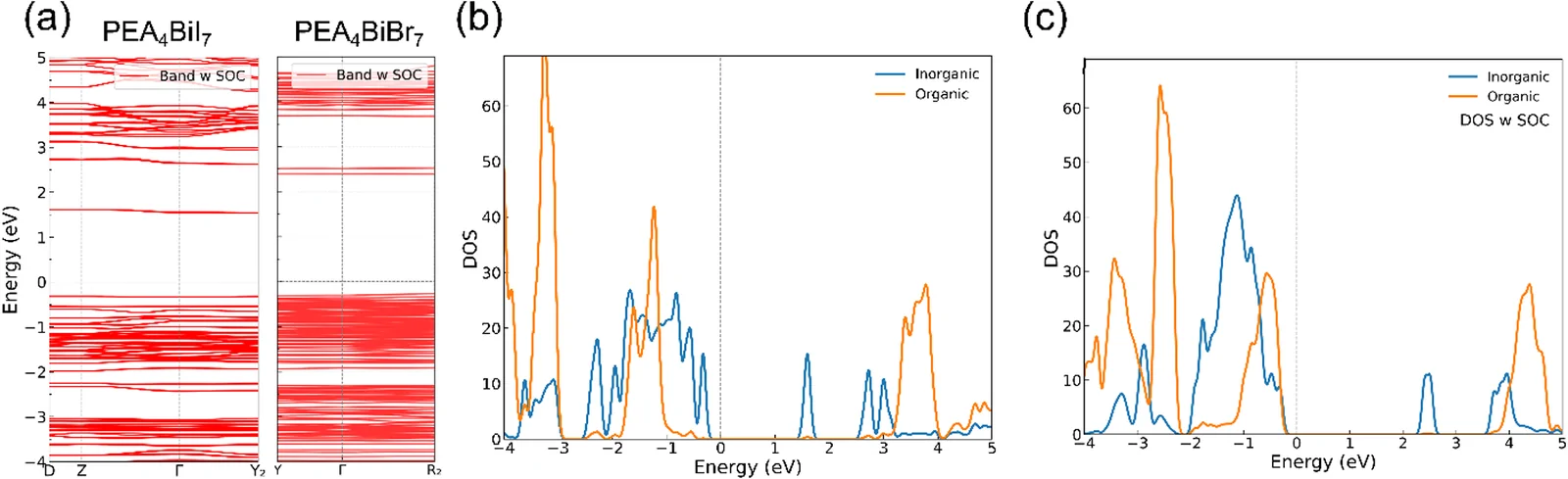
(a) Calculated band structures of PEA_4_BiBr_7_ and PEA_4_BiI_7_ with SOC. Projected densities of states of (b) PEA_4_BiI_7_ and (c) PEA_4_BiBr_7_ with SOC.

More generally, the electronic evolution from PEA_4_Bi_2_Cl_10_ to PEA_4_BiBr_7_ and PEA_4_BiI_7_ should not be interpreted as a pure halide substitution effect. It also reflects the structural change from the edge-sharing chloride bioctahedron to the isolated octahedral motifs found in the bromide and iodide. Within this limitation, the calculations support a consistent picture of highly localized bands, an inorganic conduction-band edge throughout the series, increasingly inorganic valence-band character on moving to heavier halides, and a substantial SOC contribution once Bi halide states dominate the frontier states.

## Conclusions

To conclude, we report a new class of bismuth-based organic halides incorporating PEA cations: PEA_4_BiI_7_ and PEA_4_BiBr_7_. Both compounds adopt zero-dimensional crystal structures consisting of isolated [BiX_6_]^3-^-octahedra surrounded by bulky organic cations, sharing the same stoichiometry but crystallizing in different crystal systems. These materials exhibit good stability under ambient conditions. Halide substitution plays a key role in determining the structural connectivity: while iodide and bromide systems retain isolated octahedra, the incorporation of chloride leads to a change in stoichiometry and the formation of PEA_4_Bi_2_Cl_10_, characterized by edge-sharing octahedral dimers. This structural evolution is accompanied by systematic changes in the optical properties, with the bandgap following the expected trend Cl > Br > I. Density of states calculations reveal that the conduction band edge is primarily governed by the inorganic Bi-X framework, while the valence-band edge evolves progressively from the organic cation to the inorganic sublattice across PEA_4_Bi_2_Cl_10_, PEA_4_BiBr_7_, and PEA_4_BiI_7_.

All of the compounds exhibit pronounced excitonic absorption features. Furthermore, we demonstrate that thin films can be fabricated via a simple, one-step spin-coating process. Notably, PEA_4_BiI_7_ forms highly oriented, phase-pure thin films and exhibits a broad visible PL with excitation-dependent behavior. By clarifying the complex excited-state dynamics in this system and the intrinsic tendency to self-assemble into preferentially ordered domains, we open new routes to the exploitation of the versatile chemistry of hybrid organic-inorganic Bi­(III)-based compounds, as environmentally friendly alternatives to their lead counterparts. More generally, this new family of stable, low-toxicity materials will likely provide new promising candidates for the development of next-generation solution-processable optoelectronic and electronic devices.

## Supplementary Material



## Notes

Additional experimental details, including XRD patterns of single crystals together with CIF files, TGA and DSC curves, AFM profiles, and computational methods (PDF)
