# Supplementary material for: Zero-Dimensional Bismuth Halide Thin Films with High Orientation and Broadband Emission
Source: Inorg Chem. 2026 Sep 12;65(38):22460–70. doi: 10.1021/acs.inorgchem.6c03044 (PMC13625952; doi:10.1021/acs.inorgchem.6c03044)
Supplement: Supplementary file 1 [file ic6c03044_si_001.pdf]

Supporting Information

Zero-Dimensional Bismuth Halide Thin Films with High Orientation and Broadband Emission

Raphael Neisius,[1,2] Marco Moroni,[3] Yali Yang,[4] Sergio Marras,[5] Matteo Lorenzoni,[5] Paola Ragonese,[1] Cecilia Daniela Costa,[1] Mengjiao Wang,[2] Giuseppe Ferraro,[2] Giulia Folpini,[6,7] Alessandro Stroppa,[8] Lorenzo Malavasi,[3] Teresa Gatti,[2]* Isabella Poli[1]*

1. *Istituto Italiano di Tecnologia, Center for Sustainable Future Technologies, Via Livorno 60, 10144, Torino, Italy.*
2. *Politecnico di Torino, Department of Applied Science and Technology, Corso Duca degli Abruzzi 24, 10129 Torino, Italy*
3. *Department of Chemistry and INSTM, University of Pavia, Italy*
4. *School of Mathematics and Physics, University of Science and Technology Beijing, Beijing 100083, China*
5. *Istituto Italiano di Tecnologia, Materials Characterization Facility, Via Morego 30, 16163 Genova, Italy*
6. *Consiglio Nazionale delle Ricerche, Institute for Photonics and Nanotechnology (CNR-IFN), 20133, Milano, Italy*
7. *Istituto Italiano di Tecnologia, Center for Nano Science and Technology, Via Rubattino 81, 20134, Milan, Italy*
8. *Consiglio Nazionale delle Ricerche, Institute for Superconducting and Innovative Materials and Devices (CNR-SPIN), c/o Department of Physical and Chemical Sciences, University of L'Aquila, I-67100 Coppito, L'Aquila, Italy*

Email: Isabella.poli@iit.it , teresa.gatti@polito.it

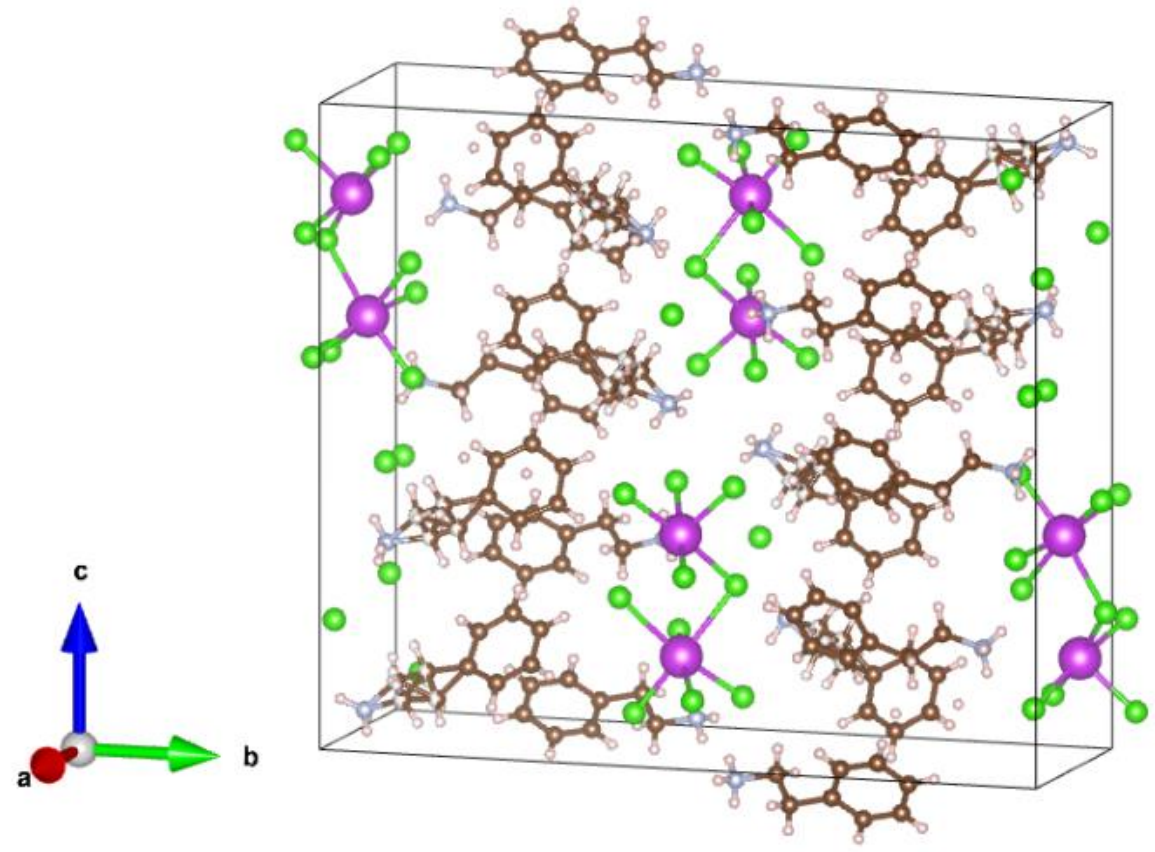

Figure S1. Three-dimensional crystal structures of $PEA_4Bi_2Cl_{10}$ determined by the single crystal X-ray diffraction results. Magenta: Bi, green: Cl, brown: C, pink: H and violet: N.

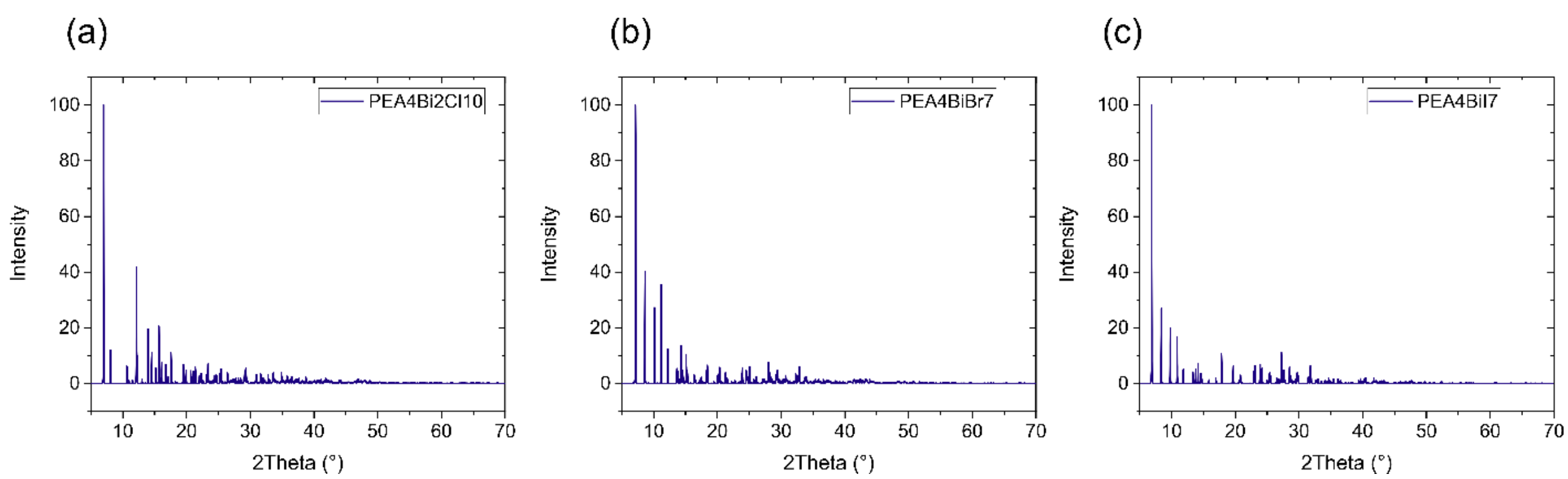

Figure S2. Calculated XRD pattern from single crystal characterization of (a) $PEA_4Bi_2Cl_{10}$, (b) PEA_4BiBr_7 and (c) PEA_4BiI_7.

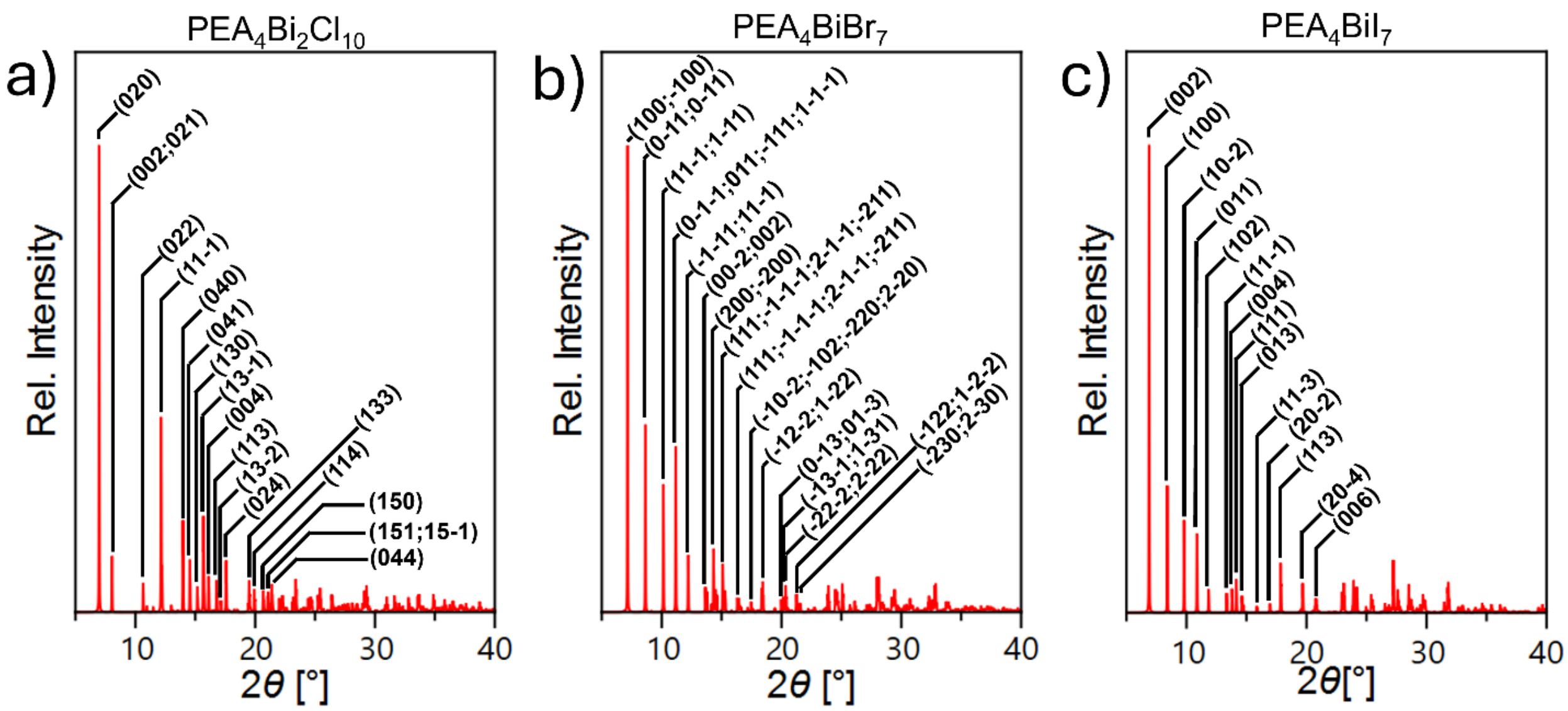

Figure S3. Calculated XRD patterns from single crystal crystallographic data of (a) $PEA_4Bi_2Cl_{10}$, (b) PEA_4BiBr_7 and (c) PEA_4BiI_7 with miller indices assigned to all dominant reflexes in the 2θ range from 5° to 25°.

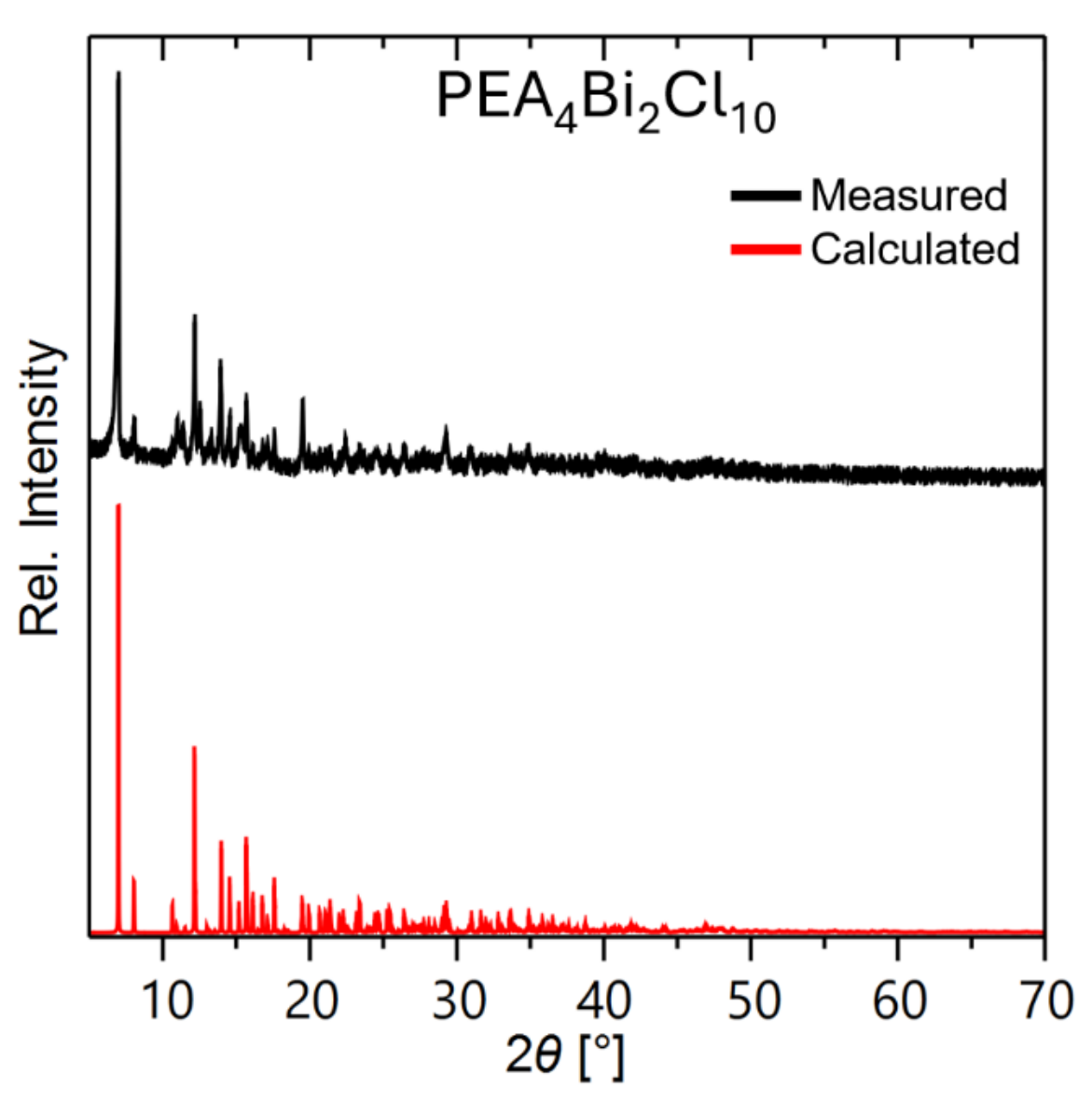

Figure S4. Calculated XRD pattern from single crystal characterization and powder XRD pattern of $PEA_4Bi_2Cl_{10}$.

Table S1. Refined crystallographic parameters obtained from Rietveld refinement of the PXRD data using PROFEX. The respective single-crystal structures were used as initial structural models.

Crystallographic Data (Powder)	$PEA_4Bi_2Cl_{10}$	PEA_4BiBr_7	PEA_4BiI_7
Source		laboratory X-ray	
Chemical Formula	$C_{32}H_{48}Bi_2Cl_{10}N_4$	$C_{32}H_{48}BiBr_7N_4$	$C_{32}H_{48}BiI_7N_4$
Formula Weight	1261.20	1257.09	1586.02
Temperature		293 K	
Wavelength for constant		1.54056 Å	
Crystal system	monoclinic	triclinic	monoclinic
Space group	P 21/c (14)	P 1 (1)	P 21/c (14)
a	8.0904 Å	13.2688 Å	10.7676 Å
b	25.4560 Å	13.4209 Å	8.6188 Å
c	22.0243 Å	13.4580 Å	26.0700 Å
α	90.000°	103.543°	90.000°
β	90.590°	92.949°	100.493°
γ	90.000°	109.207°	90.000°
V	4533.48 Å^3	2179.32 Å^3	2378.94 Å^3
Z	4	1	2
d-space range		1.3-17.6 Å	
χ^2	11.19	4.95	2.26
R_p	4.44	2.35%	3.03%
R_{wp}	5.82	3.14%	3.67%
Definition of R factors		$R_p = \sum_{i=1}^{n} \frac{\lvert y_i^{obs} - y_i^{cal}\rvert}{\sum_{i=1}^{n} y_i^{obs}}$ $R_{wp} = \left(\sum_{i=1}^{n} \frac{w_i\left(y_i^{obs} - y_i^{cal}\right)}{\sum_{i=1}^{n} w_i\left(y_i^{obs}\right)^2}\right)$	

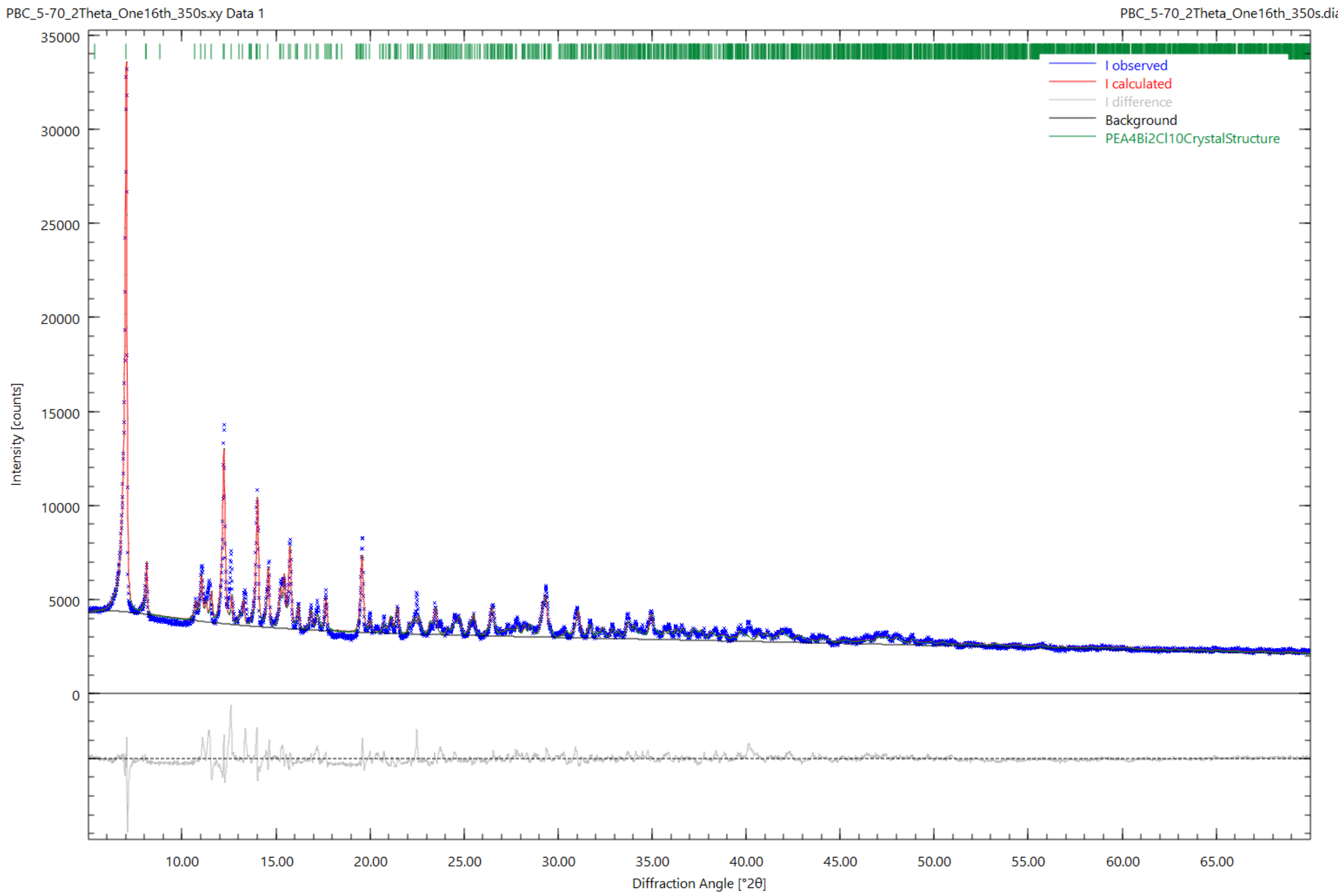

Figure S5. Rietveld refinement of the PXRD pattern of $PEA_4Bi_2Cl_{10}$ performed using PROFEX. Blue crosses represent the observed diffraction pattern, the red line the calculated profile, the black line the background, the grey line the difference (observed-calculated), and the vertical green tick marks indicate the Bragg reflection positions.

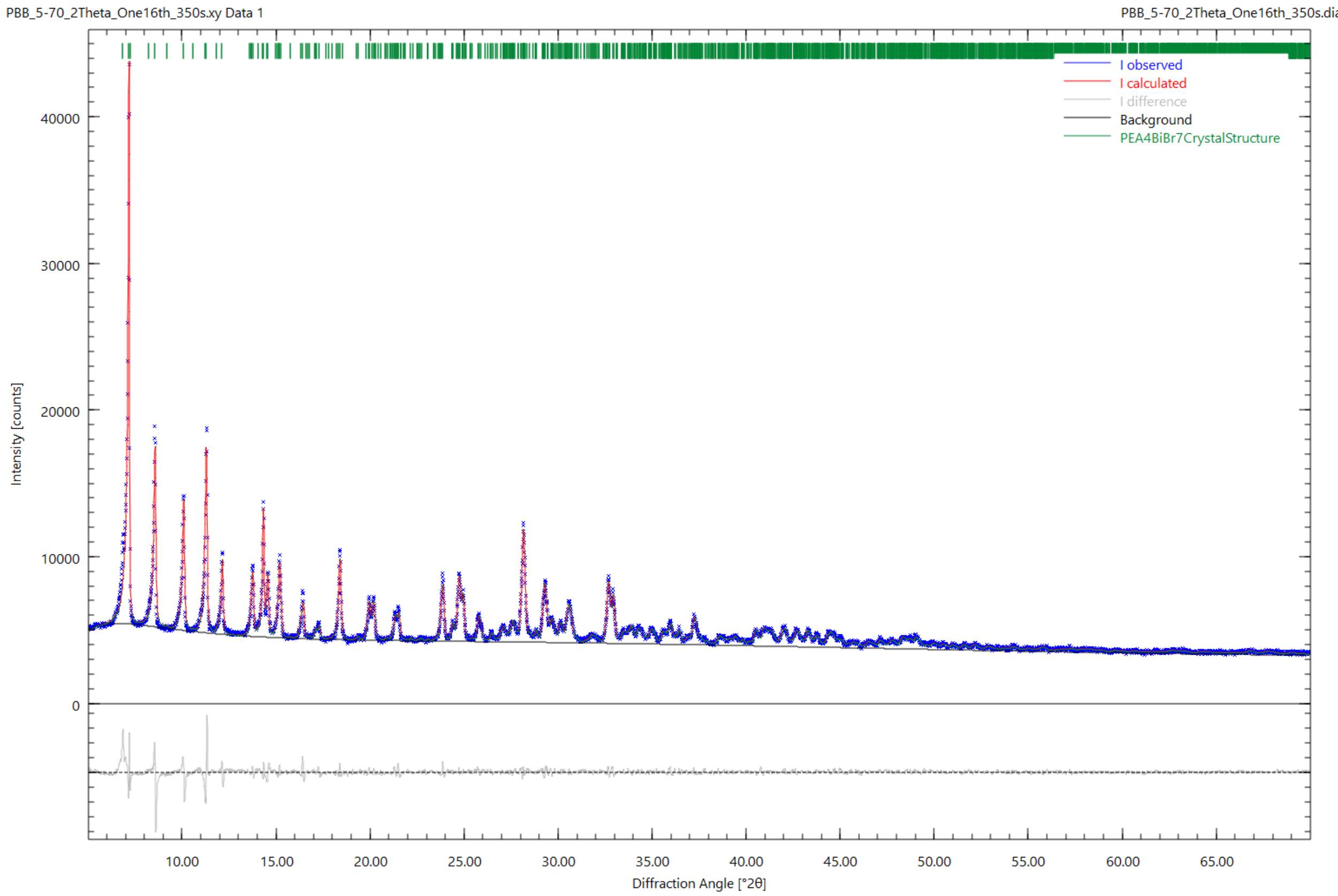

Figure S6. Rietveld refinement of the PXRD pattern of PEA_4BiBr_7 performed using PROFEX. Blue crosses represent the observed diffraction pattern, the red line the calculated profile, the black line the background, the grey line the difference (observed-calculated), and the vertical green tick marks indicate the Bragg reflection positions.

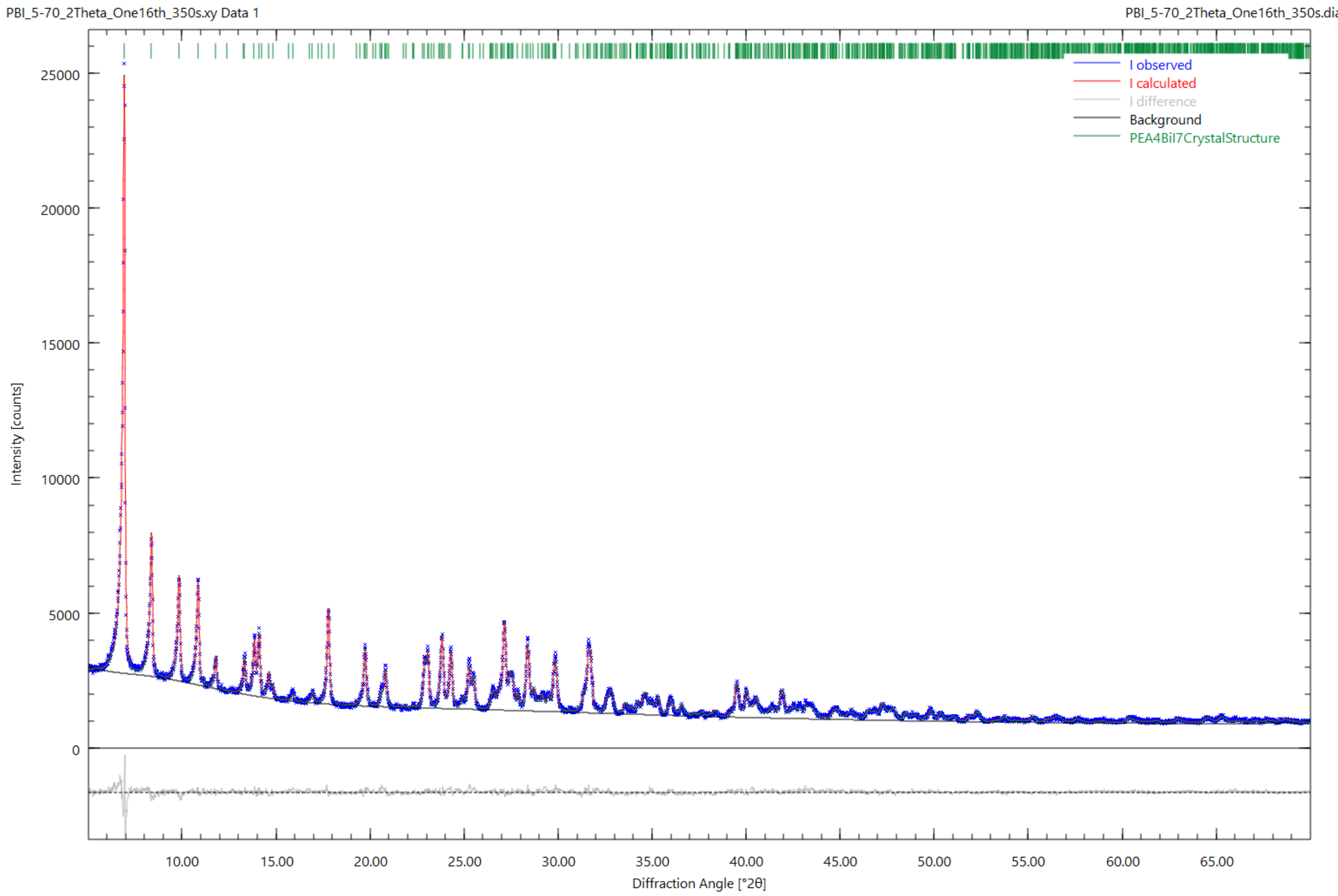

Figure S7. Rietveld refinement of the PXRD pattern of PEA_4BiI_7 performed using PROFEX. Blue crosses represent the observed diffraction pattern, the red line the calculated profile, the black line the background, the grey line the difference (observed-calculated), and the vertical green tick marks indicate the Bragg reflection positions.

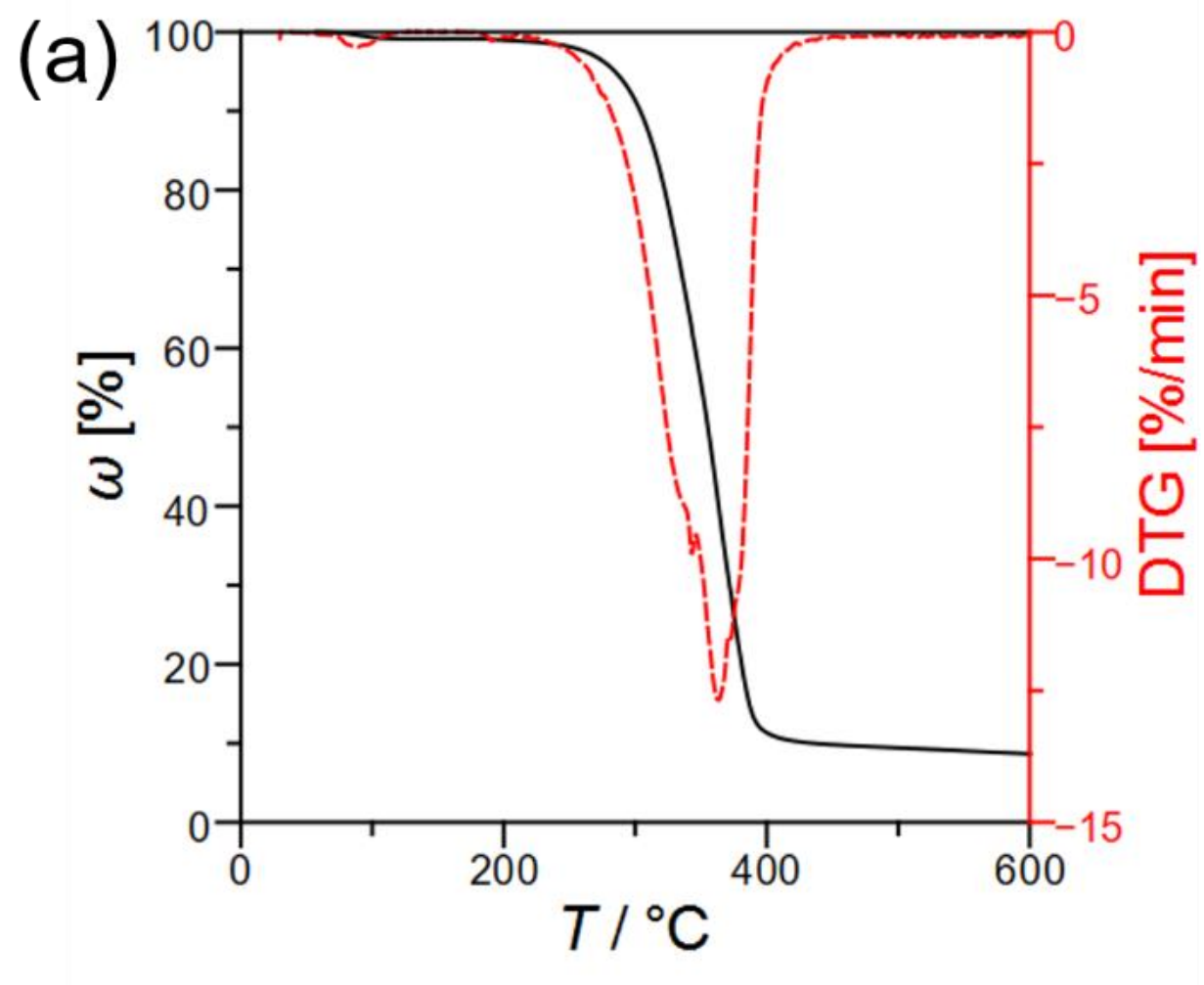

Figure S8. TGA and derivative thermogravimetry (DTG) of (a) PEA_4BiBr_7 and (b) PEA_4BiI_7 measured under nitrogen (N_2).

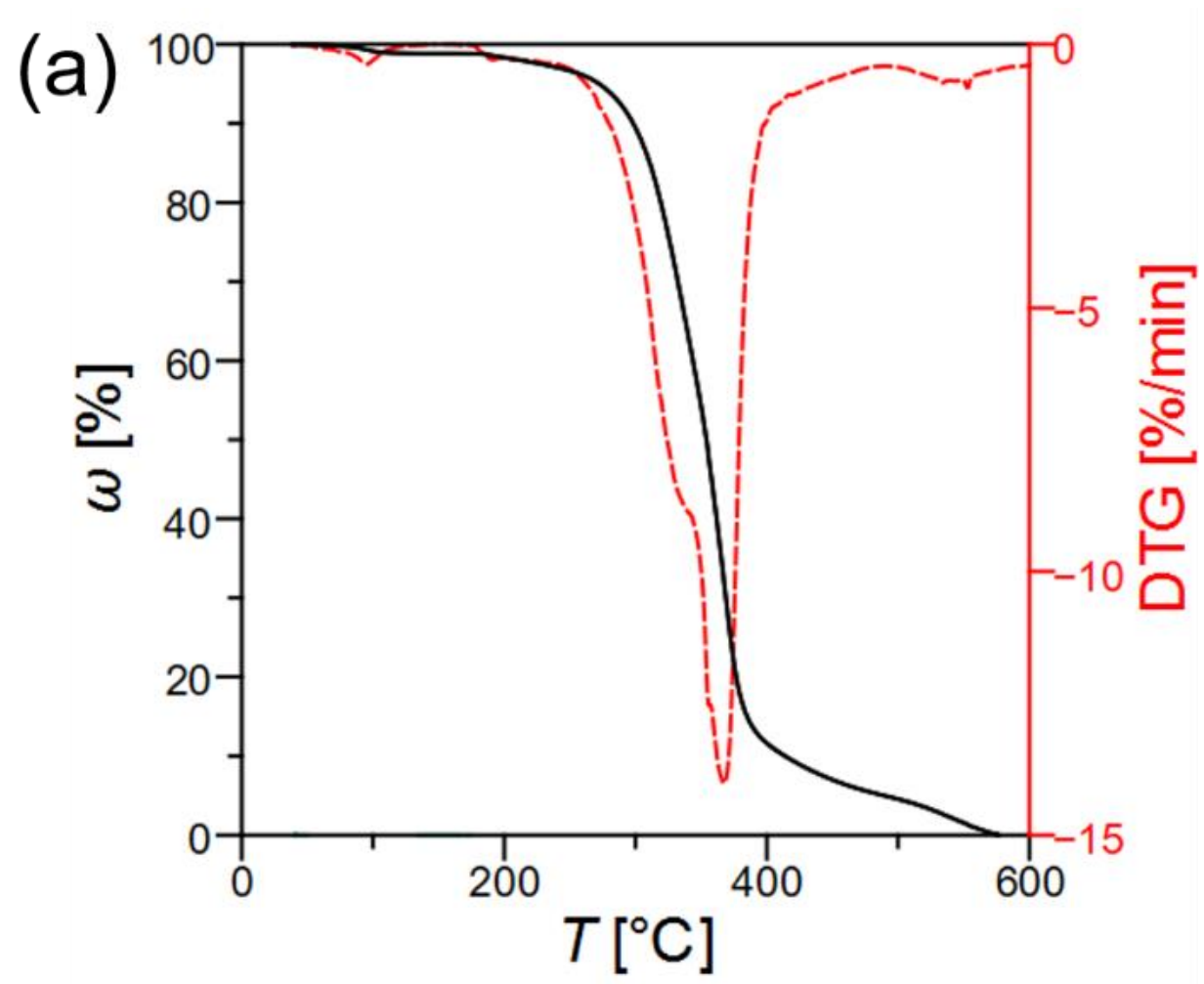

Figure S9. TGA and derivative thermogravimetry (DTG) of (a) PEA_4BiBr_7 and (b) PEA_4BiI_7 measured under air flow.

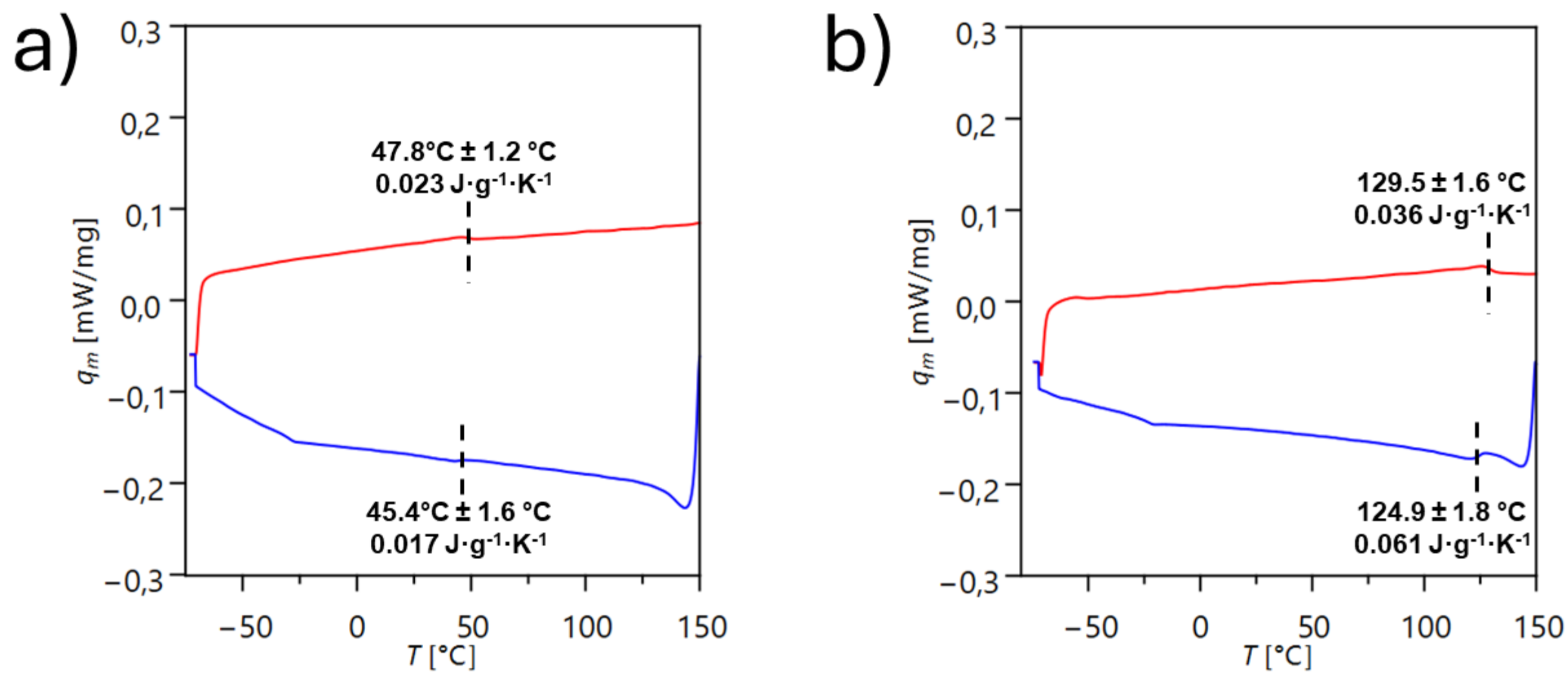

Figure S10. Differential scanning calorimetry (DSC) curves of (a) PEA_4BiBr_7 and (b) PEA_4BiI_7, recorded during the third heating (red curve) and cooling cycle (blue curve). Reversible glass transitions are observed in both materials.

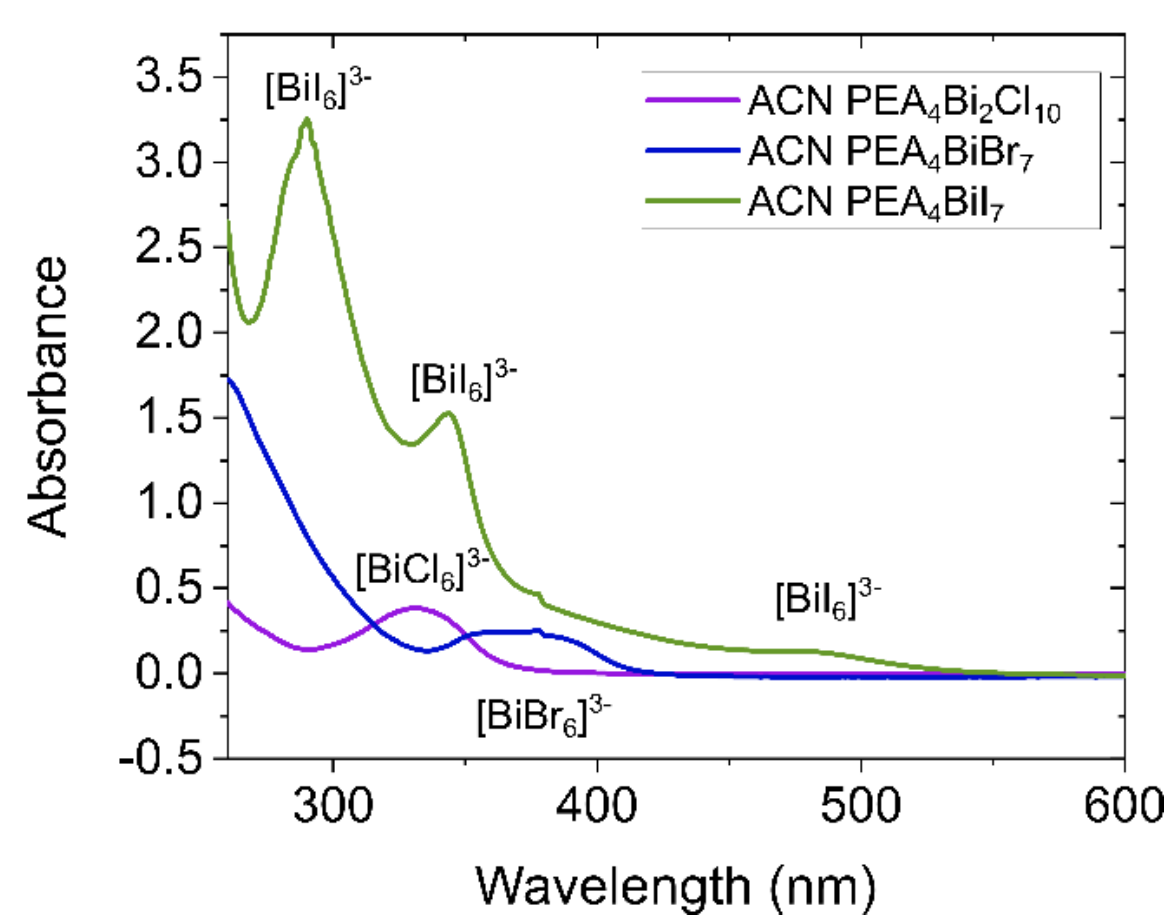

Figure S11. UV-Vis spectra of the $PEA_4Bi_2Cl_{10}$, PEA_4BiBr_7 and PEA_4BiI_7 precursor inks showing the signatures of dissolved $[BiX_6]^{3-}$-species for the respective bismuth halide component.

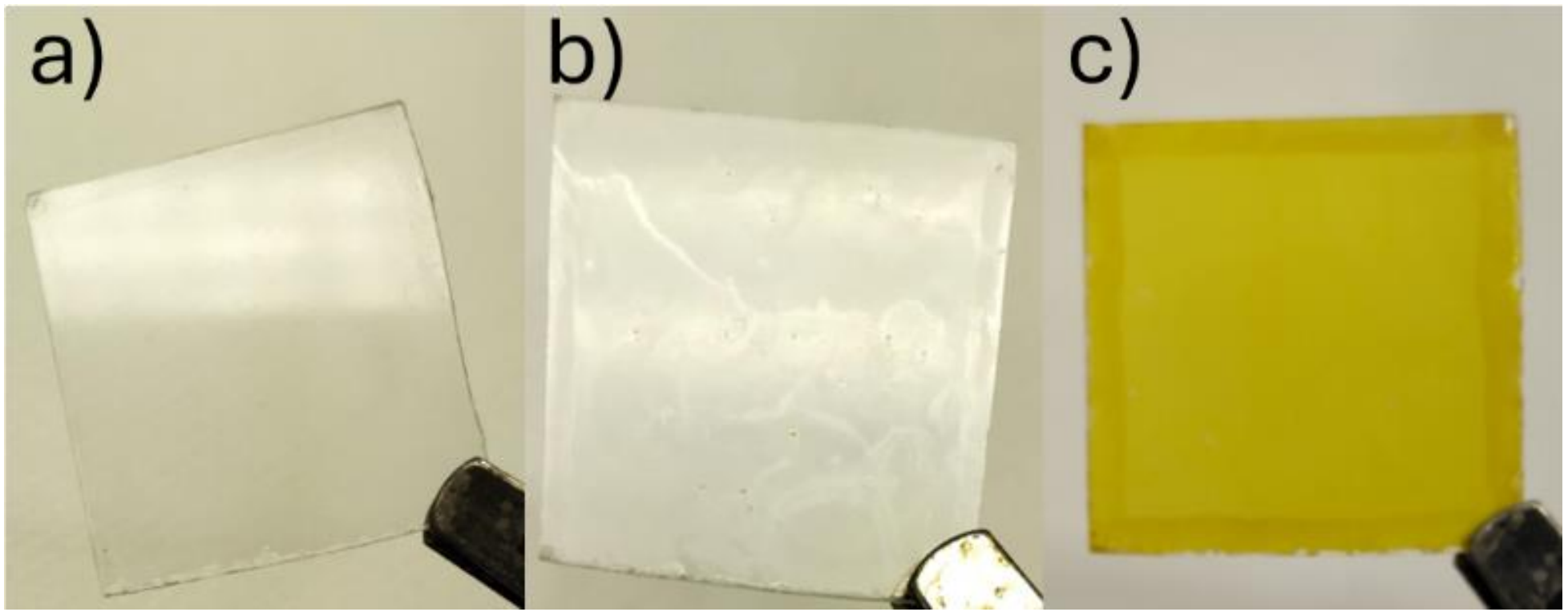

Figure S12. Pictures of spin coated thin films made for (a) $PEA_4Bi_2Cl_{10}$, (b) PEA_4BiBr_7 and (c) PEA_4BiI_7 made from the precursor inks in acetonitrile.

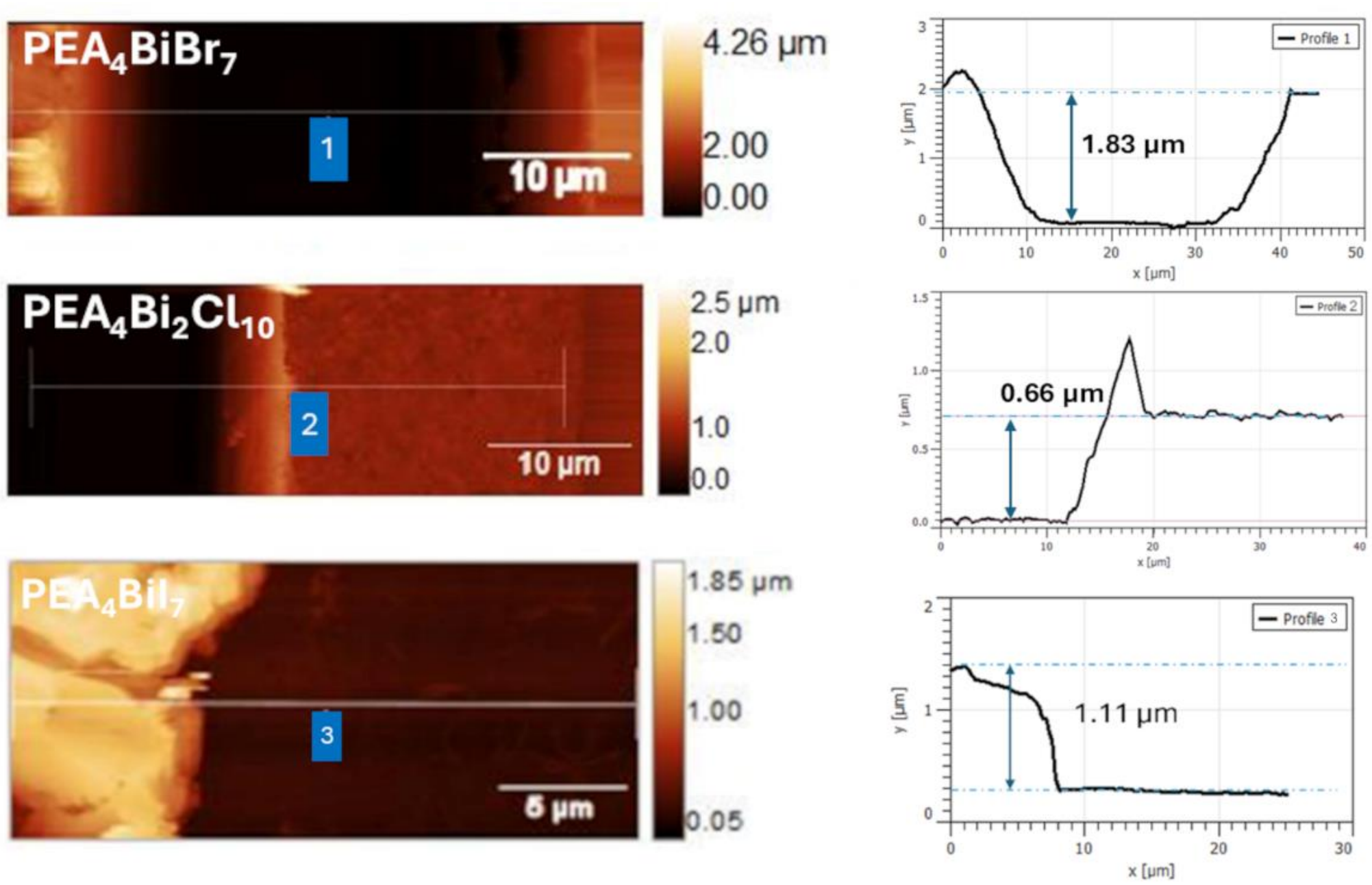

Figure S13. Example of AFM height profiles obtained from single AFM scans used to estimate film thickness. The average thickness has been calculated over three scans taken in three different spots.

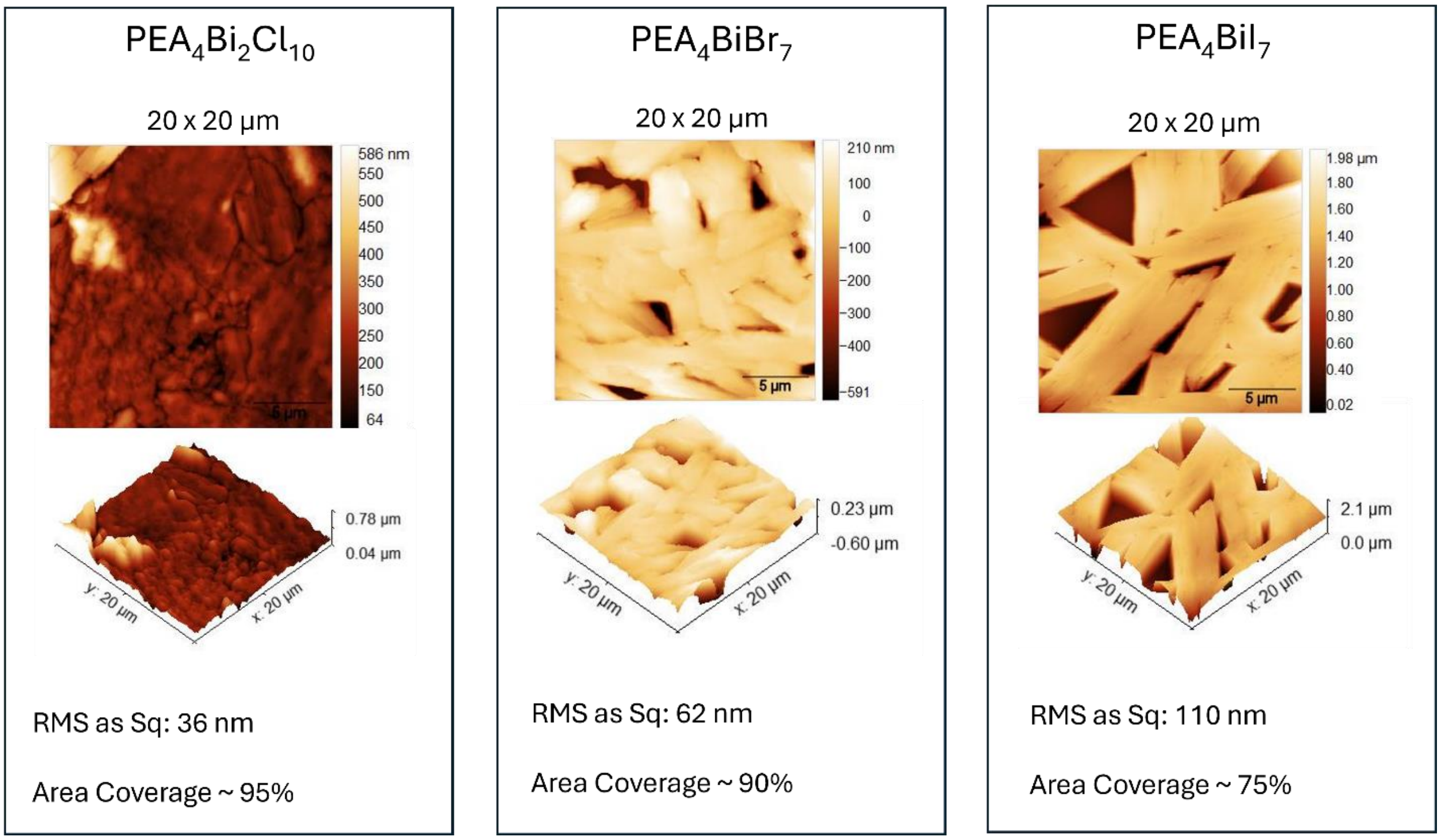

Figure S14. AFM measurements over 20 x 20 µm areas of $PEA_4Bi_2Cl_{10}$, PEA_4BiBr_7 and PEA_4BiI_7 used to extract RMS roughness and percentage area coverage.

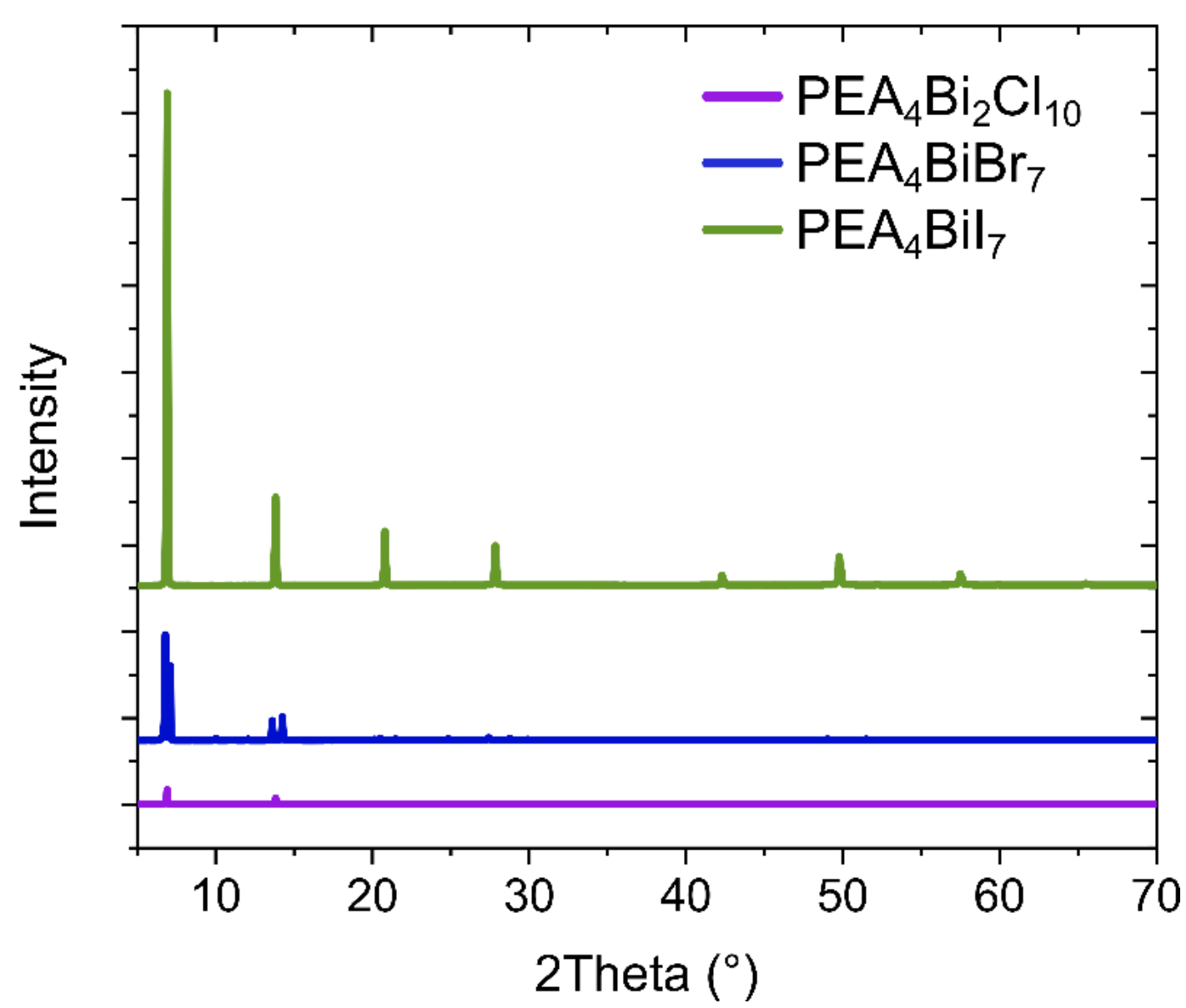

Figure S15. Absolute intensity of $PEA_4Bi_2Cl_{10}$, PEA_4BiBr_7 and PEA_4BiI_7 thin films.

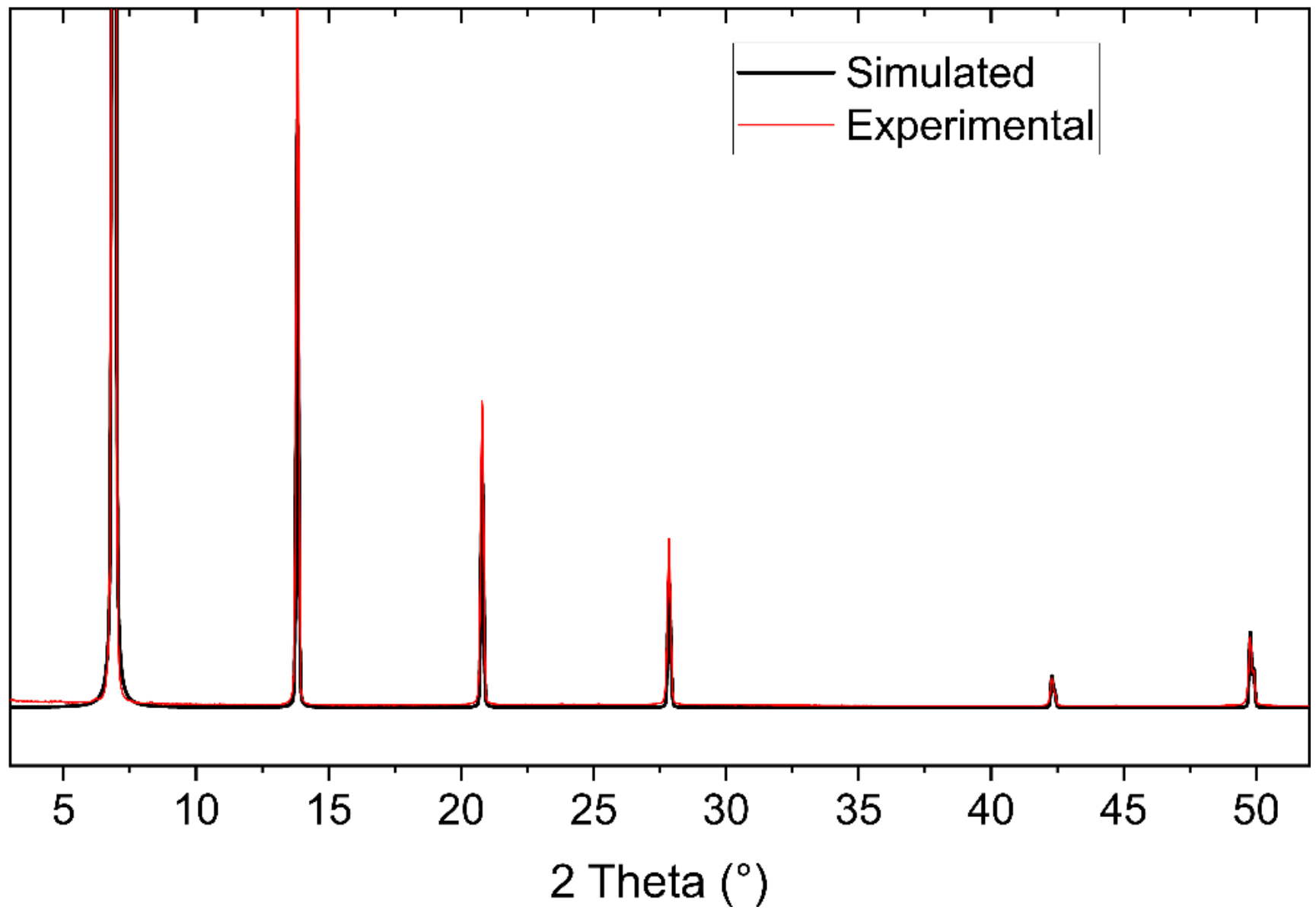

Figure S16. Experimental θ–2θ diffraction pattern of PEA_4BiI_7 and simulated diffraction pattern calculated from the reported crystal structure.

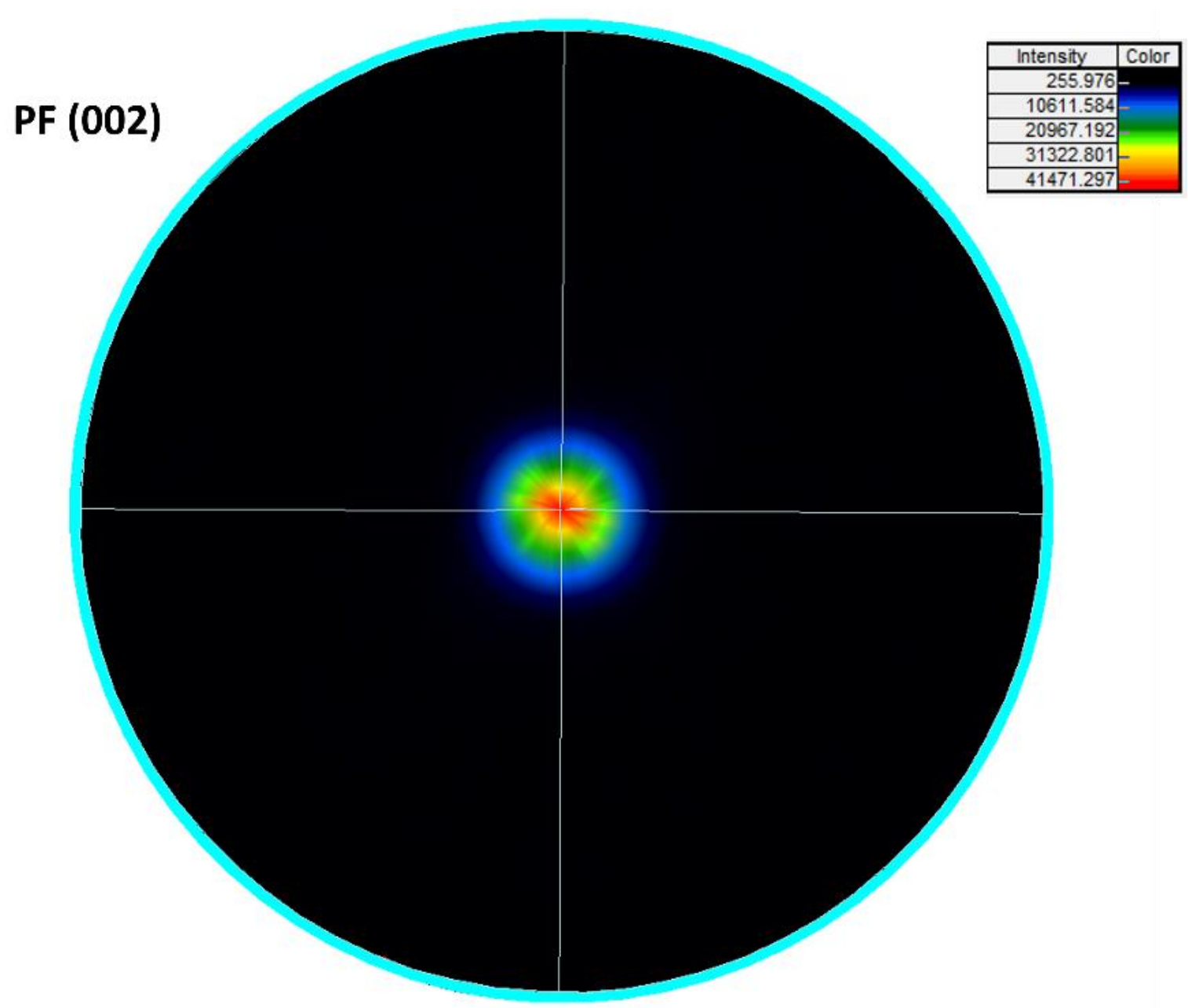

Figure S17. Top-view of the pole figure analysis of a PEA_4BiI_7 thin film based on the (002) reflection showing an azimuthally symmetric intensity distribution.

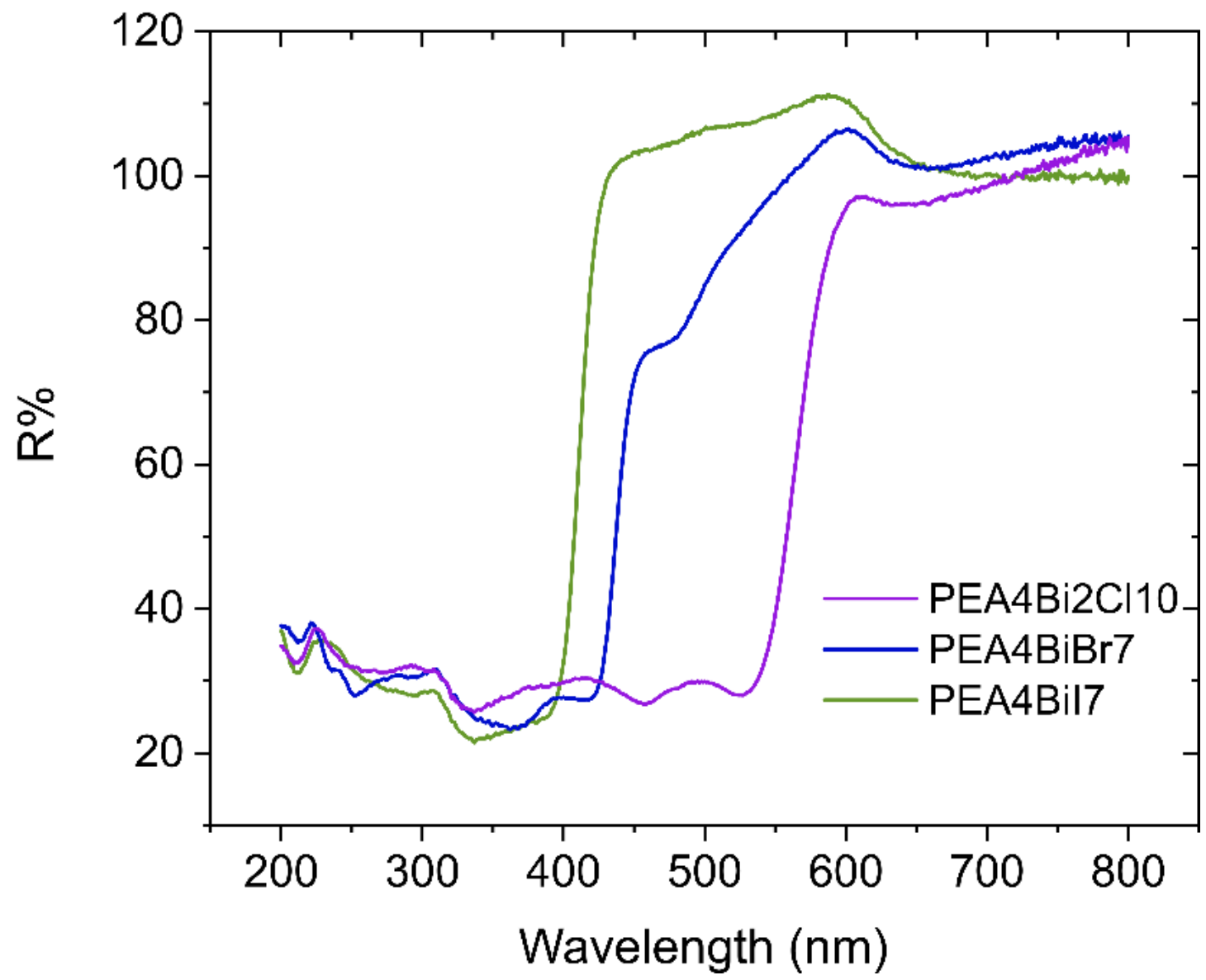

Figure S18. Diffuse reflectance spectra of pellets of $PEA_4Bi_2Cl_{10}$, PEA_4BiBr_7 and PEA_4BiI_7

FITTING PROCEDURE

These absorption near the band edge can be simulated with the Elliott model with the equation:

$$Abs(E) = A_c \cdot (1 + \mathrm{erf}(\gamma \cdot (E - E_g))) + \sum_{n=1}^{3} \frac{\beta}{n^3} \cdot \exp\left(\frac{-\left(E - E_g + \frac{E_b}{n^2}\right)^2}{\sigma^2} \right)$$

Where, E_g is the electronic band gap and E_b is the exciton binding energy. $A_c, \beta, \gamma, \sigma$ are the scaling factors. The first term describes the continuum state absorption and the second term accounts for multiple excitonic states. Only excitonic transition with $n \leq 3$ have been considered because the excitonic peak is well separated from the continuous state absorption.[1]

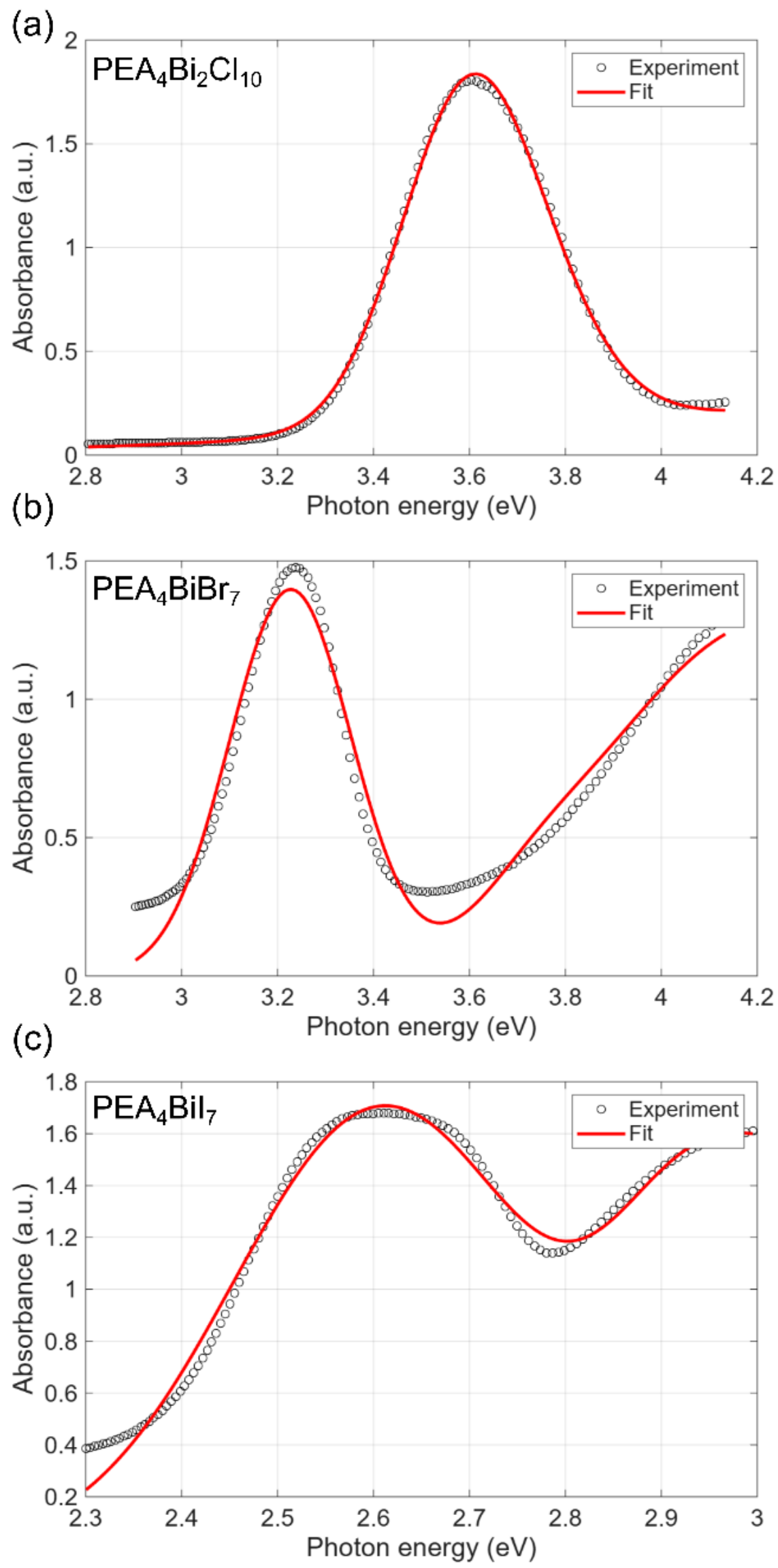

Figure S19. Cumulative fit of the near band edge absorption of (a) PEA_4Bi_2Cl10, (b) PEA_4BiBr_7 and (c) PEA_4BiI_7 with the Elliott model.

Table S2. Scaling factors extrapolated from the fitting shown in Figure S19

Parameters	$PEA_4Bi_2Cl_{10}$	PEA_4BiBr_7	PEA_4BiI_7
A_c	0.16	0.67	0.74
β	1.56	1.40	1.60
γ	0.82	3.88	10
σ	0.20	0.18	0.21

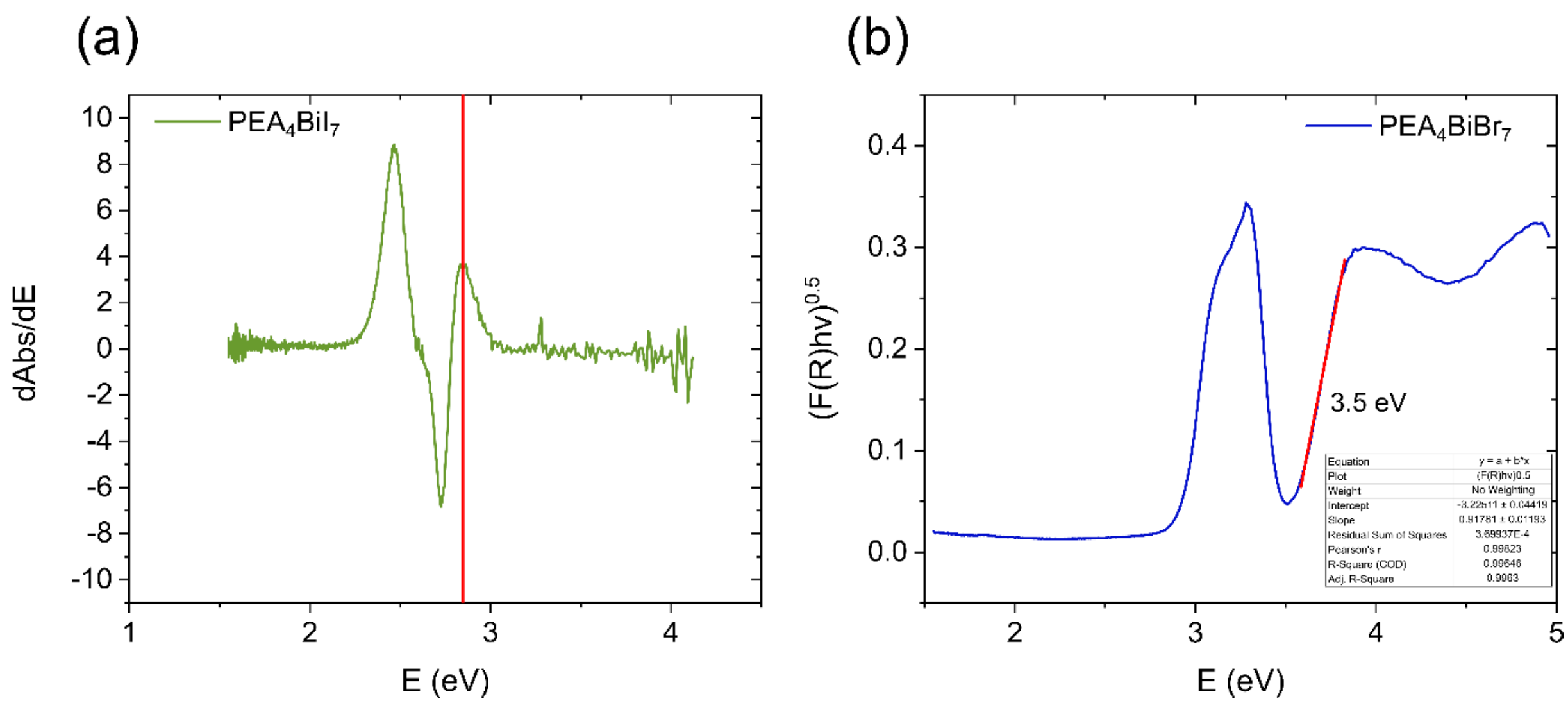

Figure S20. (a) First derivative of the absorption spectrum of PEA_4BiI_7. The higher-energy absorption edge is assigned to the fundamental bandgap (2.83 eV), while the lower energy edge corresponds to the excitonic absorption (2.46 eV). (b) Bandgap calculation from reflectance measurements assuming an indirect transition (3.5 eV).

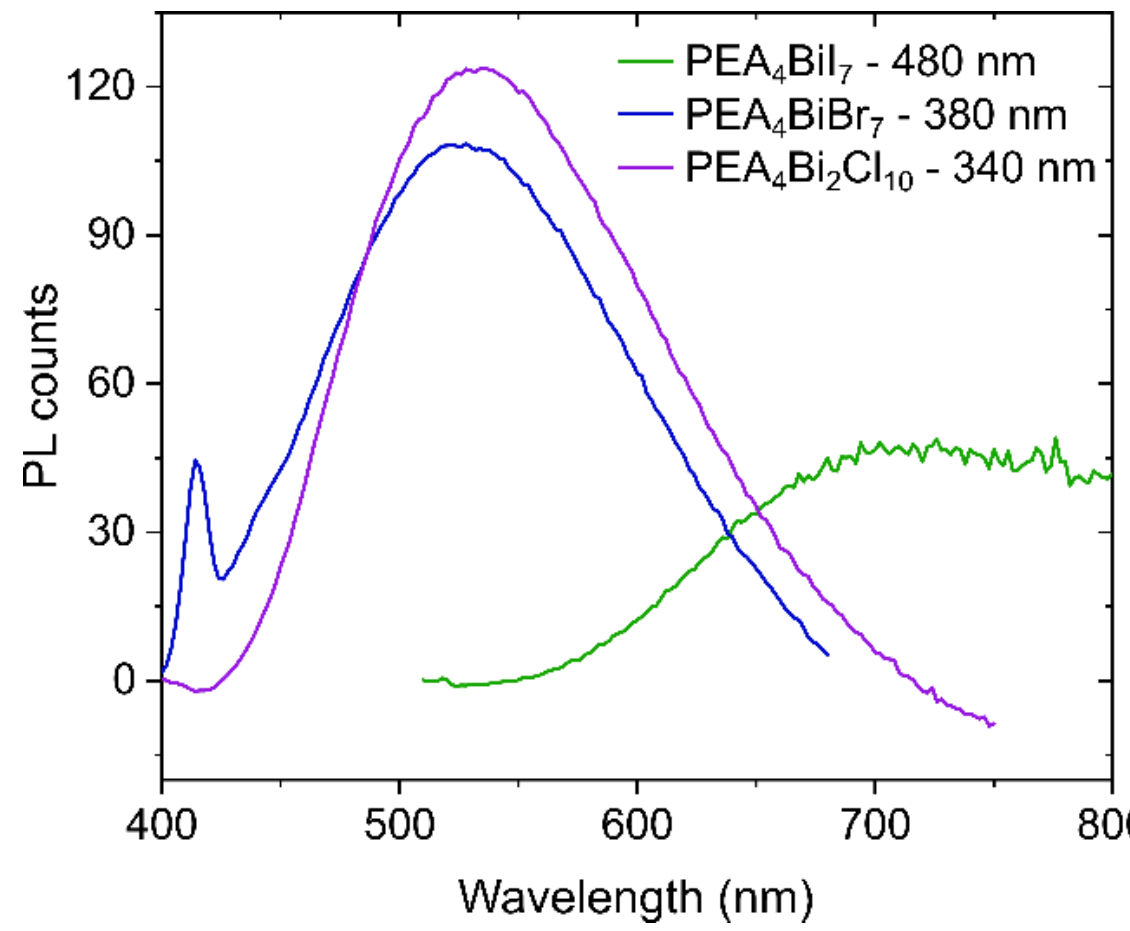

Figure S21. Photoluminescence peaks of the bismuth halide thin films excited at photon energies resonant with their excitonic absorption peaks: 340, 380, and 480 nm for $PEA_4Bi_2Cl_{10}$, PEA_4BiBr_7, and PEA_4BiI_7, respectively.

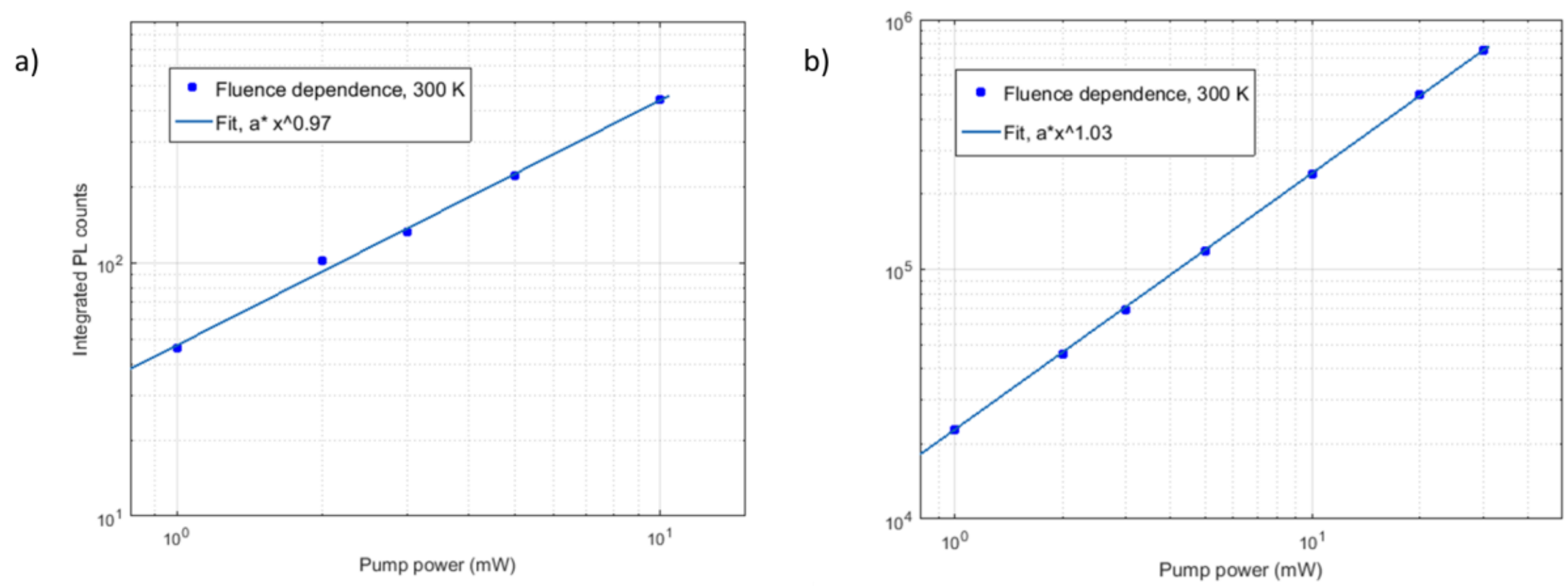

Figure S22. Fluence dependence of the photoluminescence of PEA_4BiI_7 under a) resonant excitation of the excitonic transition (pump 480 nm) or b) band gap excitation (380 nm). The measurements were performed in vacuum condition, exciting the sample with the output of an OPA at 500 kHz repetition rate, with a spot FWHM of 50 um. In both cases, the integrated PL intensity follows a power-law dependence, $I_{PL} = a * I_{pump}^{b}$, with exponents close to unity (b = 0.92 ± 0.05 for 480 nm excitation and b = 1.0028 ± 0.002 for 380 nm excitation).

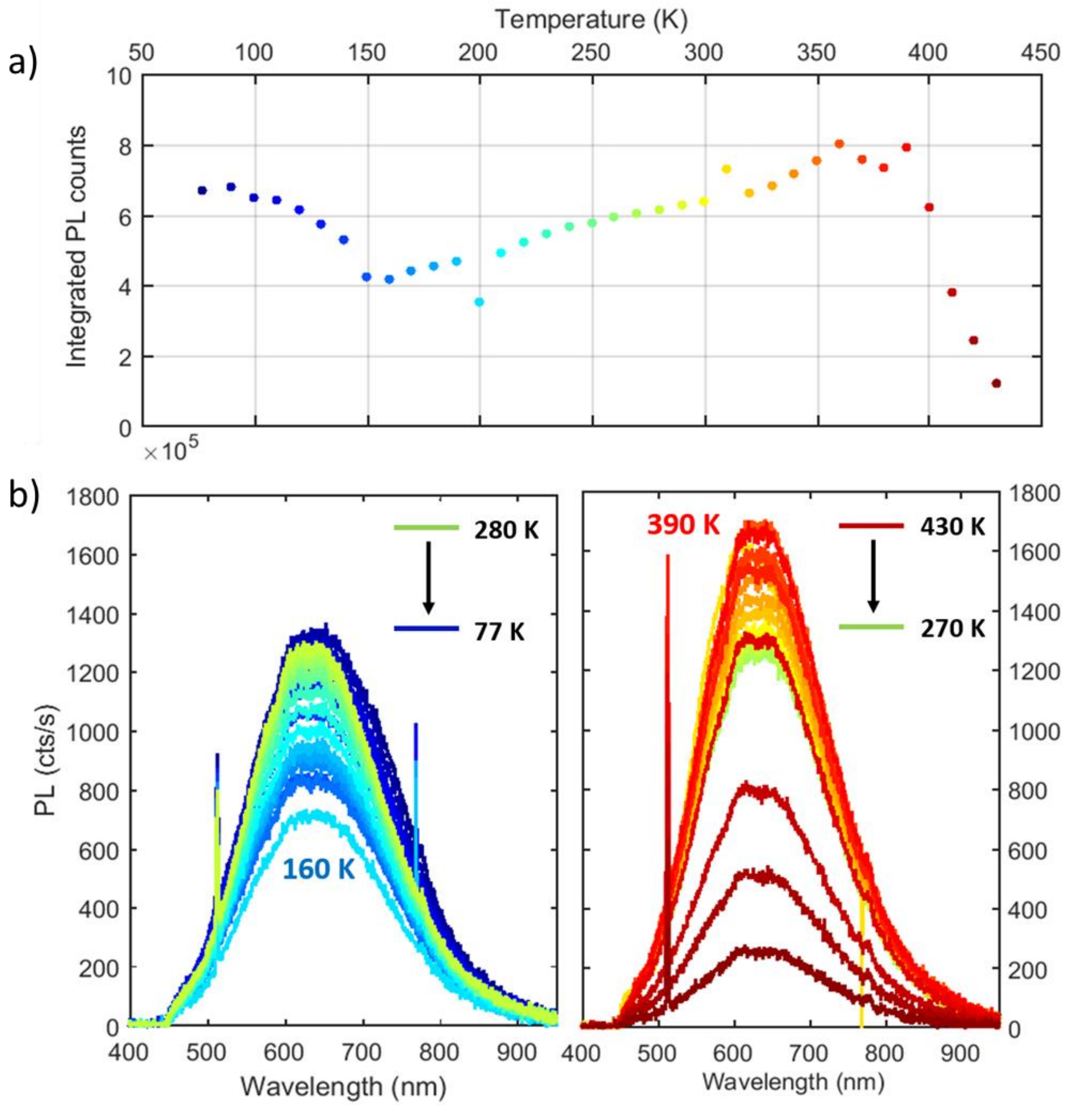

Figure S23. Temperature dependence of the PEA_4BiI_7 sample photoluminescence under excitation at 380 nm, with a fluence of 500 uJ/cm^2. a) Integrated PL intensity from 450 nm to 950 nm, b) temperature dependent spectra. A minimum in emission is found around T = 160 K. The sample emissivity increases up to room temperature and further to 390 K, where it starts sharply decreasing upon reaching the glass phase transition of the material.

COMPUTATIONAL DETAILS

SCXRD shows that the three PEA-templated bismuth halides are chemically related but not isostructural. $(PEA)_4Bi_2Cl_{10}$ crystallizes in monoclinic $P2_1/c$ and contains edge-sharing $[Bi_2Cl_{10}]^{4-}$ bioctahedra, while the bromide and iodide are both described by the reduced composition $(PEA)_4BiX_7$ (X = Br, I) and comprise isolated $[BiX_6]^{3-}$ octahedra charge-balanced by an additional halide anion. The iodide crystallizes in $P2_1/c$, whereas the bromide was refined in triclinic P1 as a two-component twin and contains two crystallographically independent Bi centers. The comparison from Cl to Br/I therefore involves both halide substitution and a change in inorganic connectivity and should not be treated as a strictly isostructural series.

Disorder is limited but important for construction of the ordered models used in the calculations. In the chloride, two terminal ethylene fragments of PEA cations are split over two 0.5/0.5 orientations (C15/C16 vs C15B/C16B and C31/C32 vs C31B/C32B, with associated H atoms). In the iodide, the only partially occupied crystallographic site is the additional iodide anion I4, refined with 0.5 occupancy in the asymmetric unit. No occupational disorder is present in the bromide atom list, although the structure was refined as a two-component twin.

Table S3. Crystallographic summary and disorder handling for the experimental and ordered structures.

**Compound**	**Experimental model**	**Disorder in CIF**	**Ordered relaxed model used for DFT**
$(PEA)_4Bi_2Cl_{10}$	$P2_1/c$; Z = 4. a = 8.0742 Å, b = 25.3398 Å, c = 21.9976 Å, β = 90.592°; V = 4500.4 Å^3.	Two split PEA fragments with 0.5/0.5 occupancies (C15/C16 and C31/C32 branches, plus H atoms).	384-atom full cell. Single branch chosen for each split fragment; experimental metric cell retained.
$(PEA)_4BiBr_7$	P1; Z = 1. a = 13.2450 Å, b = 13.2880 Å, c = 13.4205 Å, α= 102.518°, β= 93.427°, γ = 109.470°; V = 2151.5 Å^3.	No occupational disorder in the atom list. Refined as a two-component twin.	184-atom cell. Same experimental cell metrics; fully ordered coordinates.
$(PEA)_4BiI_7$	$P2_1/c$; Z = 2. a = 10.7110 Å, b = 8.5739 Å, c = 26.1410 Å, β= 100.958°; V = 2356.9 Å^3.	Additional I4 site refined at 0.5 occupancy in the asymmetric unit.	184-atom full cell. Single ordered placement chosen for the extra iodide; experimental metric cell retained.

The $(PEA)_4BiBr_7$ is reported in P1 with two independent Bi centers, while the $(PEA)_4Bi_2Cl_{10}$ and $(PEA)_4BiI_7$ phase are monoclinic. The ordered structures preserve the experimental lattice constants and only resolve partial occupancies.

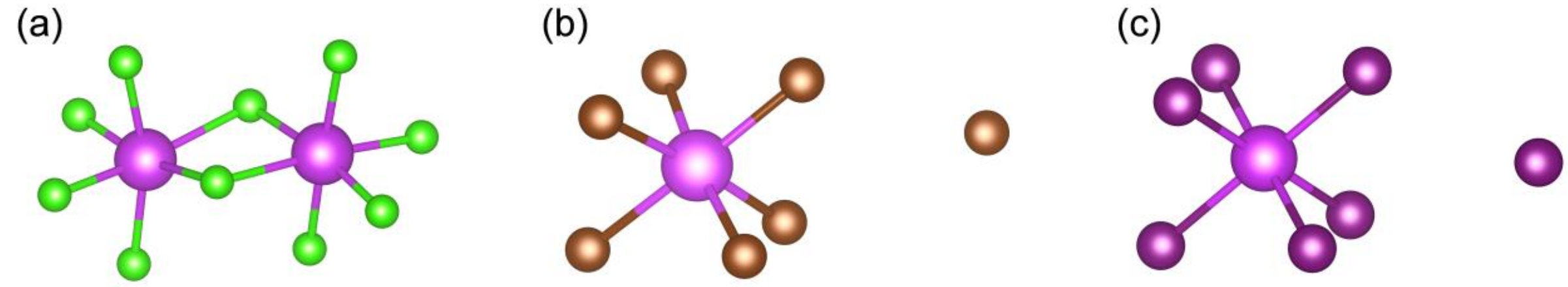

Figure S24. Local inorganic motifs in the ordered relaxed models. (a) $(PEA)_4Bi_2Cl_{10}$ contains edge-sharing $[Bi_2Cl_{10}]^{4-}$ bioctahedra, whereas (b) $(PEA)_4BiBr_7$ and (c) $(PEA)_4BiI_7$ contain isolated $[BiX_6]^{3-}$ octahedra and charge-balancing halide anions.

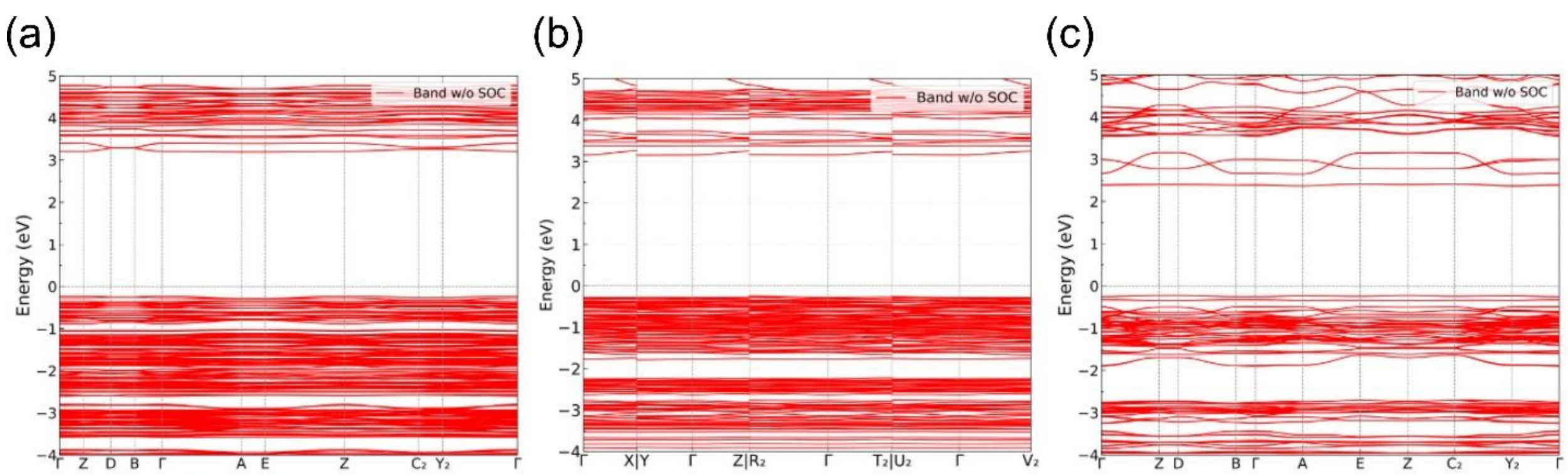

Figure S25. Calculated band structures of (a) $(PEA)_4Bi_2Cl_{10}$, (b) $(PEA)_4BiBr_7$, and (c) $(PEA)_4BiI_7$ without SOC.

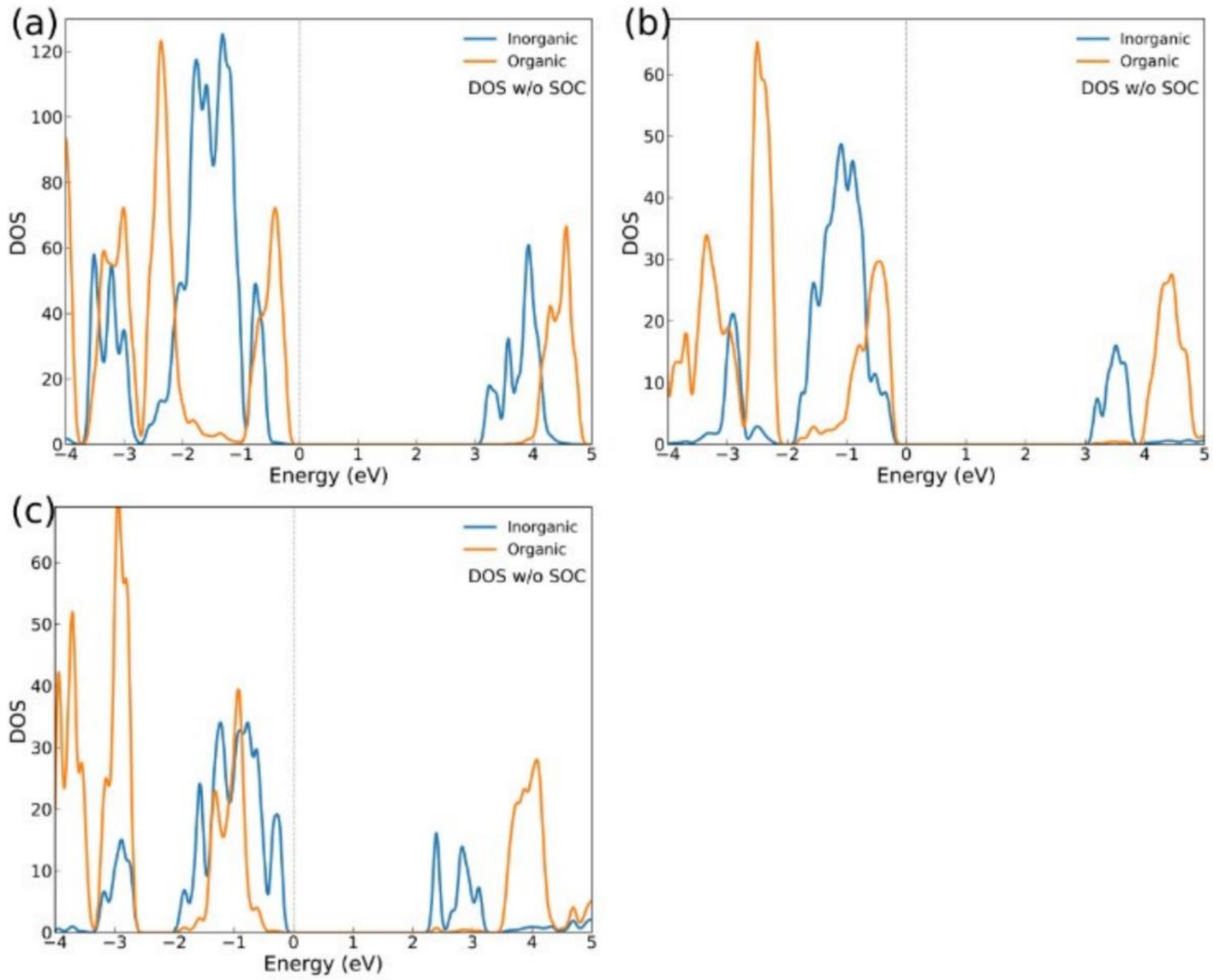

Figure S26. Projected densities of states of (a) $(PEA)_4Bi_2Cl_{10}$, (b) $(PEA)_4BiBr_7$, and (c) $(PEA)_4BiI_7$ without SOC. Across the series, the valence-band edge evolves from a stronger organic contribution in $(PEA)_4Bi_2Cl_{10}$ toward increasingly inorganic character in $(PEA)_4BiBr_7$ and especially $(PEA)_4BiI_7$, whereas the conduction-band edge remains predominantly inorganic.

REFINED ATOMIC COORDINATES

Table S4. Refined atomic coordinates from single crystal data for $PEA_4Bi_2Cl_{10}$.

No.	Atom	*x*	*y*	*z*	Occ.	*U*
1	Bi1	0.22736	0.52050	0.65278	1.000	0.044
2	Bi2	0.33686	0.53104	0.84844	1.000	0.050
3	Cl1	0.50490	0.55592	0.58952	1.000	0.058
4	Cl2	0.20960	0.44414	0.57356	1.000	0.054
5	Cl3	0.03610	0.57864	0.58113	1.000	0.055
6	Cl4	-0.05520	0.48570	0.69864	1.000	0.078
7	Cl5	0.20850	0.60374	0.75344	1.000	0.052
8	Cl6	0.42550	0.46391	0.73949	1.000	0.059
9	Cl7	0.04860	0.48488	0.89697	1.000	0.068
10	Cl8	0.63890	0.56650	0.82818	1.000	0.081
11	Cl9	0.49330	0.46181	0.91633	1.000	0.062
12	Cl10	0.32150	0.59507	0.93870	1.000	0.077
13	C1	0.86040	0.74670	0.70340	1.000	0.072
14	C2	0.78270	0.78550	0.67100	1.000	0.067
15	H2	0.72511	0.77602	0.63584	1.000	0.081
16	C3	0.78470	0.83650	0.68710	1.000	0.093
17	H3	0.72956	0.86168	0.66375	1.000	0.112
18	C4	0.87140	0.85080	0.73970	1.000	0.149
19	H4	0.88181	0.88613	0.75055	1.000	0.181
20	C5	0.93900	0.81300	0.77430	1.000	0.122
21	H5	0.98738	0.82215	0.81145	1.000	0.147
22	C6	0.94010	0.76240	0.75690	1.000	0.088
23	H6	0.99444	0.73747	0.78085	1.000	0.106
24	C7	0.85840	0.69100	0.68140	1.000	0.096
25	H7A	0.82441	0.69069	0.63897	1.000	0.115
26	H7B	0.97000	0.67698	0.68384	1.000	0.115
27	C8	0.75010	0.65700	0.71490	1.000	0.108
28	H8A	0.63731	0.66962	0.70995	1.000	0.130
29	H8B	0.77871	0.65950	0.75779	1.000	0.130
30	N1	0.75680	0.60030	0.69620	1.000	0.082
31	H1A	0.68705	0.58155	0.71867	1.000	0.124
32	H1B	0.72796	0.59743	0.65714	1.000	0.124
33	H1C	0.85921	0.58804	0.70154	1.000	0.124
34	C9	0.34270	0.82980	0.88260	1.000	0.092
35	C10	0.44990	0.82570	0.83450	1.000	0.083
36	H10	0.50383	0.85615	0.82185	1.000	0.100
37	C11	0.48090	0.78150	0.80520	1.000	0.080
38	H11	0.55278	0.78144	0.77247	1.000	0.096
39	C12	0.40720	0.73580	0.82310	1.000	0.091
40	H12	0.42888	0.70425	0.80313	1.000	0.109
41	C13	0.30050	0.73710	0.87120	1.000	0.090

42	H13	0.24765	0.70633	0.88342	1.000	0.108
43	C14	0.27200	0.78200	0.90030	1.000	0.113
44	H14	0.20254	0.78167	0.93379	1.000	0.136
**No.**	**Atom**	***x***	***y***	***z***	**Occ.**	***U***
45	C15	0.32000	0.87060	0.93360	0.500	0.182
46	H15A	0.24173	0.85765	0.96329	0.500	0.221
47	H15B	0.42479	0.87731	0.95413	0.500	0.221
48	C16	0.25500	0.92090	0.90430	0.500	0.176
49	H16A	0.33335	0.93312	0.87423	0.500	0.207
50	H16B	0.15078	0.91366	0.88371	0.500	0.207
51	N2	0.23080	0.96240	0.95060	1.000	0.081
52	H2A	0.19281	0.99155	0.93265	1.000	0.121
53	H2B	0.15789	0.95133	0.97784	1.000	0.121
54	H2C	0.32687	0.96934	0.96906	1.000	0.121
55	C15B	0.36300	0.88940	0.89500	0.500	0.167
56	H15C	0.46097	0.89587	0.91977	0.500	0.199
57	H15D	0.37543	0.90842	0.85673	0.500	0.199
58	C16B	0.21200	0.90710	0.92960	0.500	0.160
59	H16C	0.11727	0.90525	0.90206	0.500	0.192
60	H16D	0.19054	0.88387	0.96372	0.500	0.192
61	C17	0.12360	0.29830	1.03980	1.000	0.066
62	C18	0.20270	0.26290	1.07550	1.000	0.085
63	H18	0.25724	0.27498	1.11026	1.000	0.102
64	C19	0.20630	0.20990	1.06300	1.000	0.083
65	H19	0.26269	0.18673	1.08866	1.000	0.100
66	C20	0.12660	0.19180	1.01280	1.000	0.085
67	H20	0.12983	0.15611	1.00311	1.000	0.102
68	C21	0.04210	0.22590	0.97680	1.000	0.078
69	H21	-0.01608	0.21324	0.94312	1.000	0.094
70	C22	0.04130	0.27760	0.98900	1.000	0.076
71	H22	-0.01559	0.30032	0.96304	1.000	0.091
72	C23	0.11930	0.35680	1.05400	1.000	0.108
73	H23A	0.00677	0.36971	1.04909	1.000	0.130
74	H23B	0.15267	0.36235	1.09598	1.000	0.130
75	C24	0.22680	0.38640	1.01500	1.000	0.076
76	H24A	0.19130	0.38107	0.97317	1.000	0.092
77	H24B	0.33819	0.37236	1.01908	1.000	0.092
78	N3	0.23150	0.44410	1.02770	1.000	0.072
79	H3A	0.30025	0.45976	1.00193	1.000	0.109
80	H3B	0.13038	0.45758	1.02301	1.000	0.109
81	H3C	0.26676	0.44950	1.06563	1.000	0.109
82	C25	0.68090	0.29950	0.88330	1.000	0.096
83	C26	0.58790	0.29750	0.93470	1.000	0.088
84	H26	0.56078	0.32917	0.95358	1.000	0.106
85	C27	0.53420	0.25330	0.95920	1.000	0.092
86	H27	0.48111	0.25350	0.99649	1.000	0.110

87	C28	0.55850	0.20680	0.92830	1.000	0.108
88	H28	0.51616	0.17547	0.94376	1.000	0.130
89	C29	0.64270	0.20610	0.87600	1.000	0.088
**No.**	**Atom**	***x***	***y***	***z***	**Occ.**	***U***
90	H29	0.66093	0.17446	0.85566	1.000	0.106
91	C30	0.70130	0.25240	0.85300	1.000	0.095
92	H30	0.75621	0.25217	0.81605	1.000	0.114
93	C31	0.77420	0.35080	0.87320	0.500	0.054
94	H31A	0.76371	0.37352	0.90839	0.500	0.065
95	H31B	0.89081	0.34361	0.86701	0.500	0.065
96	C32	0.70100	0.37750	0.81760	0.500	0.052
97	H32A	0.72363	0.35661	0.78173	0.500	0.063
98	H32B	0.58193	0.38044	0.82176	0.500	0.063
99	N4	0.77450	0.43080	0.81090	1.000	0.083
100	H4A	0.73126	0.44651	0.77817	1.000	0.125
101	H4B	0.75260	0.45003	0.84373	1.000	0.125
102	H4C	0.88364	0.42799	0.80671	1.000	0.125
103	C31B	0.67300	0.34140	0.83230	0.500	0.103
104	H31C	0.71276	0.32756	0.79403	0.500	0.122
105	H31D	0.55997	0.35348	0.82669	0.500	0.122
106	C32B	0.78100	0.38720	0.85490	0.500	0.101
107	H32C	0.74403	0.39911	0.89444	0.500	0.118
108	H32D	0.89418	0.37476	0.85927	0.500	0.118

Table S5. Anisotropic thermal displacement parameters from single crystal data for $PEA_4Bi_2Cl_{10}$.

Label	U^{11}	U^{22}	U^{33}	U^{23}	U^{13}	U^{12}
Bi1	0.04998(19)	0.04441(19)	0.03802(18)	0.00101(12)	-0.00083(13)	0.00268(12)
Bi2	0.0593(2)	0.0519(2)	0.03944(19)	0.00234(13)	-0.00203(14)	0.00494(14)
Cl1	0.0468(11)	0.0497(12)	0.0777(16)	0.0018(11)	0.0200(10)	-0.0005(9)
Cl2	0.0666(13)	0.0420(11)	0.0541(12)	-0.0174(9)	-0.0002(10)	-0.0003(9)
Cl3	0.0657(13)	0.0585(13)	0.0394(10)	0.0130(9)	0.0019(9)	0.0208(10)
Cl4	0.0730(17)	0.0704(17)	0.092(2)	0.0264(15)	0.0340(15)	0.0035(13)
Cl5	0.0982(16)	0.0210(7)	0.0347(9)	-0.0046(6)	-0.0312(10)	0.0162(9)
Cl6	0.0716(15)	0.0537(12)	0.0516(13)	-0.0087(10)	-0.0226(11)	0.0228(11)
Cl7	0.0466(12)	0.093(2)	0.0659(16)	0.0086(14)	0.0039(11)	-0.0042(12)
Cl8	0.0736(17)	0.107(2)	0.0616(15)	0.0142(15)	-0.0012(13)	-0.0315(16)
Cl9	0.0668(15)	0.0655(15)	0.0541(13)	0.0168(11)	0.0078(11)	0.0148(11)
Cl10	0.100(2)	0.0740(17)	0.0579(15)	-0.0319(13)	-0.0176(14)	0.0237(15)
C1	0.052(6)	0.080(8)	0.083(8)	-0.008(6)	0.016(5)	0.005(5)
C2	0.063(6)	0.075(7)	0.064(6)	-0.011(6)	0.007(5)	0.000(5)
C3	0.073(8)	0.087(10)	0.120(12)	0.026(8)	0.031(8)	-0.004(7)
C4	0.088(12)	0.131(16)	0.23(3)	-0.078(17)	0.072(14)	-0.064(11)
C5	0.094(12)	0.20(2)	0.071(9)	-0.041(12)	0.006(8)	-0.052(13)
C6	0.064(7)	0.127(12)	0.074(8)	0.006(8)	-0.006(6)	-0.015(7)

C7	0.108(10)	0.071(8)	0.110(11)	-0.003(8)	0.042(8)	0.011(7)
C8	0.113(11)	0.071(9)	0.140(13)	0.010(9)	0.048(10)	0.019(8)
N1	0.101(7)	0.064(6)	0.083(7)	0.003(5)	0.028(6)	0.011(5)
**Label**	U^{11}	U^{22}	U^{33}	U^{23}	U^{13}	U^{12}
C9	0.107(10)	0.074(8)	0.094(10)	-0.013(7)	0.012(8)	0.004(7)
C10	0.089(9)	0.082(9)	0.079(8)	0.025(7)	-0.020(7)	-0.015(7)
C11	0.078(8)	0.105(10)	0.057(7)	-0.008(7)	0.021(5)	-0.012(7)
C12	0.091(9)	0.092(10)	0.089(9)	-0.031(8)	0.006(7)	0.012(8)
C13	0.073(8)	0.074(8)	0.122(12)	0.018(8)	0.008(7)	-0.003(6)
C14	0.112(12)	0.134(15)	0.094(11)	0.007(10)	0.055(9)	0.014(10)
C15	0.32(4)	0.12(3)	0.11(2)	0.04(2)	0.14(3)	0.00(3)
C16	0.26(4)	0.11(3)	0.16(3)	0.04(2)	0.10(3)	-0.04(2)
N2	0.118(9)	0.056(5)	0.069(6)	-0.008(4)	0.031(6)	-0.010(5)
C15B	0.25(3)	0.043(14)	0.21(4)	-0.03(2)	0.05(3)	0.04(2)
C16B	0.31(4)	0.062(17)	0.11(2)	-0.006(17)	0.07(3)	0.05(2)
C17	0.075(7)	0.062(6)	0.062(6)	-0.011(5)	0.017(5)	-0.021(5)
C18	0.082(8)	0.109(10)	0.065(7)	-0.008(7)	-0.015(6)	-0.033(7)
C19	0.074(7)	0.094(9)	0.081(8)	0.020(7)	-0.006(6)	0.000(7)
C20	0.103(9)	0.067(7)	0.087(9)	0.005(7)	0.028(7)	-0.023(7)
C21	0.086(8)	0.093(9)	0.055(6)	-0.010(6)	0.002(6)	-0.033(7)
C22	0.083(8)	0.073(8)	0.071(7)	0.010(6)	-0.007(6)	-0.006(6)
C23	0.130(12)	0.084(9)	0.112(11)	-0.020(9)	0.061(9)	-0.018(9)
C24	0.114(9)	0.054(6)	0.061(6)	-0.006(5)	0.005(6)	-0.009(6)
N3	0.089(6)	0.066(6)	0.062(5)	0.002(4)	0.003(5)	-0.001(5)
C25	0.106(9)	0.052(6)	0.131(12)	0.023(7)	0.071(9)	0.021(6)
C26	0.072(7)	0.078(8)	0.116(11)	-0.028(8)	0.029(7)	-0.011(6)
C27	0.081(8)	0.109(11)	0.086(9)	0.023(9)	-0.011(7)	-0.017(8)
C28	0.096(10)	0.081(10)	0.148(15)	0.063(10)	-0.010(10)	-0.008(8)
C29	0.100(10)	0.059(7)	0.105(11)	-0.016(7)	-0.016(8)	0.018(7)
C30	0.089(9)	0.118(12)	0.078(9)	0.009(8)	0.020(7)	0.050(8)
C31	0.037(8)	0.064(11)	0.060(10)	0.001(9)	-0.027(8)	-0.011(8)
C32	0.048(10)	0.063(12)	0.046(10)	0.003(8)	-0.015(8)	0.002(9)
N4	0.104(8)	0.067(6)	0.078(6)	0.029(5)	0.014(5)	0.001(5)
C31B	0.068(15)	0.13(2)	0.11(2)	-0.005(19)	-0.015(15)	0.005(15)
C32B	0.074(16)	0.11(2)	0.12(2)	0.009(16)	0.040(15)	-0.008(13)

Table S6. Refined atomic coordinates from single crystal data for PEA_4BiBr_7.

No.	Atom	*x*	*y*	*z*	Occ.	*U*
1	C1	0.44000	0.71300	0.10200	1.000	0.069
2	H1A	0.40993	0.75859	0.14848	1.000	0.079
3	H1B	0.42574	0.64416	0.12244	1.000	0.079
4	C2	0.38600	0.68900	-0.00800	1.000	0.104
5	H2A	0.41278	0.63946	-0.05398	1.000	0.125
6	H2B	0.40439	0.75722	-0.03013	1.000	0.125
7	C3	0.26300	0.63700	-0.01600	1.000	0.089
8	C4	0.21400	0.52400	-0.06100	1.000	0.090
9	H4	0.25640	0.48208	-0.08256	1.000	0.114
10	C5	0.10300	0.47600	-0.07300	1.000	0.113
11	H5	0.07058	0.40038	-0.10081	1.000	0.135
12	C6	0.03800	0.53700	-0.04400	1.000	0.121
13	H6	-0.03690	0.50536	-0.05453	1.000	0.145
14	C7	0.09000	0.64800	0.00300	1.000	0.103
15	H7	0.04865	0.69101	0.02318	1.000	0.121
16	C8	0.20200	0.69900	0.02000	1.000	0.107
17	H8	0.23486	0.77342	0.05414	1.000	0.127
18	N1	0.55600	0.76900	0.11000	1.000	0.073
19	H1C	0.58705	0.78262	0.17441	1.000	0.110
20	H1D	0.58359	0.72651	0.06779	1.000	0.110
21	H1E	0.56897	0.83236	0.09188	1.000	0.110
22	C9	0.40600	0.12300	0.09800	1.000	0.110
23	H9A	0.39674	0.04587	0.07415	1.000	0.135
24	H9B	0.36802	0.14222	0.04520	1.000	0.135
25	C10	0.35700	0.14000	0.19700	1.000	0.146
26	H10A	0.35795	0.21534	0.21661	1.000	0.175
27	H10B	0.39856	0.12834	0.25170	1.000	0.175
28	C11	0.23900	0.05900	0.18000	1.000	0.118
29	C12	0.15600	0.08500	0.13900	1.000	0.100
30	H12	0.17010	0.15046	0.11872	1.000	0.118
31	C13	0.05000	0.01200	0.12600	1.000	0.122
32	H13	-0.00609	0.02818	0.09615	1.000	0.150
33	C14	0.02500	-0.08400	0.15800	1.000	0.090
34	H14	-0.04562	-0.13201	0.15256	1.000	0.108
35	C15	0.11200	-0.10600	0.19900	1.000	0.144
36	H15	0.09690	-0.16977	0.22237	1.000	0.176
37	C16	0.21900	-0.03900	0.20700	1.000	0.123
38	H16	0.27462	-0.06041	0.22856	1.000	0.147
39	N2	0.52000	0.18900	0.11200	1.000	0.062

40	H2C	0.54579	0.17630	0.05197	1.000	0.094
41	H2D	0.52944	0.26011	0.13206	1.000	0.094
42	H2E	0.55606	0.17079	0.15890	1.000	0.094
43	C17	0.81700	0.12200	0.81500	1.000	0.140
**No.**	**Atom**	***x***	***y***	***z***	**Occ.**	***U***
44	H17A	0.83682	0.16719	0.76651	1.000	0.170
45	H17B	0.82058	0.05029	0.78490	1.000	0.170
46	C18	0.89500	0.17600	0.91600	1.000	0.158
47	H18A	0.88027	0.12716	0.96142	1.000	0.187
48	H18B	0.88584	0.24393	0.94973	1.000	0.187
49	C19	1.01200	0.20100	0.89400	1.000	0.117
50	C20	1.06700	0.13500	0.91400	1.000	0.106
51	H20	1.03043	0.06937	0.93119	1.000	0.125
52	C21	1.17700	0.16500	0.90900	1.000	0.123
53	H21	1.21362	0.12010	0.92413	1.000	0.148
54	C22	1.23400	0.26100	0.88200	1.000	0.160
55	H22	1.30661	0.27950	0.87512	1.000	0.192
56	C23	1.17600	0.32700	0.86600	1.000	0.161
57	H23	1.21404	0.39470	0.85252	1.000	0.198
58	C24	1.06600	0.29900	0.86800	1.000	0.155
59	H24	1.02960	0.34360	0.85274	1.000	0.184
60	N3	0.70700	0.11000	0.83600	1.000	0.117
61	H3A	0.66181	0.07804	0.77698	1.000	0.183
62	H3B	0.68923	0.06751	0.87977	1.000	0.183
63	H3C	0.70425	0.17561	0.86277	1.000	0.183
64	C25	0.87700	0.15800	0.25000	1.000	0.126
65	H25A	0.87640	0.12277	0.17887	1.000	0.147
66	H25B	0.92279	0.13659	0.29348	1.000	0.147
67	C26	0.92200	0.28300	0.26600	1.000	0.143
68	H26A	0.91172	0.31823	0.33387	1.000	0.170
69	H26B	0.88284	0.30345	0.21521	1.000	0.170
70	C27	1.04300	0.32400	0.25600	1.000	0.098
71	C28	1.12000	0.33500	0.33500	1.000	0.131
72	H28	1.09948	0.31233	0.39428	1.000	0.160
73	C29	1.23000	0.38000	0.32700	1.000	0.124
74	H29	1.28142	0.39045	0.38197	1.000	0.148
75	C30	1.26200	0.40900	0.23800	1.000	0.179
76	H30	1.33472	0.43121	0.22843	1.000	0.216
77	C31	1.18200	0.40400	0.16400	1.000	0.143
78	H31	1.20319	0.43085	0.10734	1.000	0.171
79	C32	1.07300	0.36000	0.17000	1.000	0.122
80	H32	1.02112	0.35406	0.11654	1.000	0.143
81	N4	0.76700	0.12300	0.27600	1.000	0.095
82	H4A	0.74199	0.05031	0.26620	1.000	0.141
83	H4B	0.76811	0.15531	0.34147	1.000	0.141
84	H4C	0.72518	0.14253	0.23540	1.000	0.141

85	C33	0.45100	0.15800	0.54300	1.000	0.065
86	H33A	0.42817	0.12183	0.59732	1.000	0.077
87	H33B	0.42926	0.10239	0.47752	1.000	0.077
88	C34	0.39700	0.24400	0.54100	1.000	0.081
**No.**	**Atom**	***x***	***y***	***z***	**Occ.**	***U***
89	H34A	0.42641	0.30452	0.60176	1.000	0.095
90	H34B	0.41218	0.27284	0.48058	1.000	0.095
91	C35	0.27500	0.19000	0.53700	1.000	0.075
92	C36	0.20700	0.15500	0.44500	1.000	0.131
93	H36	0.23659	0.16761	0.38517	1.000	0.155
94	C37	0.09700	0.10100	0.43800	1.000	0.153
95	H37	0.05340	0.07870	0.37495	1.000	0.184
96	C38	0.05200	0.07900	0.52400	1.000	0.110
97	H38	-0.02171	0.04037	0.52095	1.000	0.137
98	C39	0.12200	0.11800	0.61600	1.000	0.113
99	H39	0.09211	0.10778	0.67609	1.000	0.134
100	C40	0.23300	0.17000	0.62500	1.000	0.091
101	H40	0.27694	0.19160	0.68846	1.000	0.110
102	N5	0.56790	0.21500	0.56000	1.000	0.063
103	H5A	0.60061	0.16677	0.56188	1.000	0.092
104	H5B	0.58777	0.24784	0.50979	1.000	0.092
105	H5C	0.58677	0.26580	0.62042	1.000	0.092
106	C41	0.90500	0.70200	0.76300	1.000	0.094
107	H41A	0.91783	0.69753	0.69203	1.000	0.113
108	H41B	0.93638	0.65511	0.78943	1.000	0.113
109	C42	0.96100	0.82100	0.82700	1.000	0.116
110	H42A	0.94881	0.82661	0.89782	1.000	0.137
111	H42B	0.93069	0.86910	0.80011	1.000	0.137
112	C43	1.08300	0.85800	0.82100	1.000	0.085
113	C44	1.15400	0.85400	0.89900	1.000	0.089
114	H44	1.12702	0.82952	0.95516	1.000	0.106
115	C45	1.26400	0.88600	0.89400	1.000	0.103
116	H45	1.31032	0.88483	0.94789	1.000	0.127
117	C46	1.30600	0.92100	0.81000	1.000	0.106
118	H46	1.37855	0.93544	0.80142	1.000	0.130
119	C47	1.23300	0.93200	0.73900	1.000	0.139
120	H47	1.26063	0.96993	0.69055	1.000	0.168
121	C48	1.12200	0.89000	0.73500	1.000	0.111
122	H48	1.07547	0.88334	0.67746	1.000	0.131
123	N6	0.78900	0.66300	0.76600	1.000	0.094
124	H6A	0.75938	0.59333	0.72836	1.000	0.146
125	H6B	0.77701	0.66557	0.83099	1.000	0.146
126	H6C	0.75983	0.70489	0.74074	1.000	0.146
127	C49	0.37400	0.59300	0.57100	1.000	0.100
128	H49A	0.35686	0.51444	0.54881	1.000	0.125
129	H49B	0.35184	0.61804	0.51347	1.000	0.125

No.	Atom	*x*	*y*	*z*	Occ.	*U*
130	C50	0.31100	0.61900	0.66000	1.000	0.105
131	H50A	0.34134	0.60600	0.72176	1.000	0.125
132	H50B	0.31740	0.69539	0.67444	1.000	0.125
133	C51	0.19100	0.54400	0.63100	1.000	0.087
**No.**	**Atom**	***x***	***y***	***z***	**Occ.**	***U***
134	C52	0.11700	0.58900	0.60200	1.000	0.099
135	H52	0.13952	0.66207	0.59838	1.000	0.116
136	C53	0.00800	0.52300	0.57700	1.000	0.121
137	H53	-0.04214	0.55435	0.55932	1.000	0.151
138	C54	-0.02900	0.41300	0.57900	1.000	0.128
139	H54	-0.10086	0.36764	0.55725	1.000	0.157
140	C55	0.04800	0.37500	0.61400	1.000	0.129
141	H55	0.02432	0.30498	0.62550	1.000	0.155
142	C56	0.15800	0.43600	0.63400	1.000	0.108
143	H56	0.20755	0.40394	0.64915	1.000	0.131
144	N7	0.49000	0.64800	0.60300	1.000	0.081

Table S7. Anisotropic thermal displacement parameters from single crystal data for PEA_4BiBr_7

Label	U^{11}	U^{22}	U^{33}	U^{23}	U^{13}	U^{12}
C1	0.056(19)	0.04(2)	0.09(3)	0.001(19)	-0.010(18)	0.003(17)
C2	0.11(2)	0.11(4)	0.10(3)	0.03(3)	-0.01(2)	0.05(2)
C3	0.10(2)	0.08(2)	0.09(4)	0.02(2)	-0.03(2)	0.043(19)
C4	0.13(3)	0.08(2)	0.06(3)	0.01(2)	0.02(3)	0.04(2)
C5	0.13(3)	0.10(3)	0.08(3)	0.00(3)	0.00(3)	0.02(2)
C6	0.10(3)	0.17(5)	0.07(4)	0.01(4)	-0.03(3)	0.04(3)
C7	0.12(3)	0.14(4)	0.08(3)	0.04(3)	0.00(3)	0.08(3)
C8	0.13(3)	0.08(3)	0.10(4)	0.00(3)	0.00(4)	0.04(3)
N1	0.062(18)	0.06(3)	0.10(3)	0.01(2)	0.013(18)	0.030(18)
C9	0.06(3)	0.07(3)	0.15(5)	0.00(3)	0.06(3)	-0.03(2)
C10	0.08(2)	0.18(5)	0.13(4)	0.04(4)	0.04(3)	-0.02(3)
C11	0.06(2)	0.15(4)	0.16(6)	0.09(4)	0.05(3)	0.02(2)
C12	0.10(3)	0.07(3)	0.12(4)	0.02(3)	0.02(3)	0.02(2)
C13	0.07(2)	0.16(5)	0.15(5)	0.06(4)	0.05(3)	0.04(3)
C14	0.06(2)	0.07(3)	0.10(4)	-0.03(2)	0.03(2)	0.00(2)
C15	0.10(3)	0.17(6)	0.16(6)	0.11(5)	0.03(4)	0.00(3)
C16	0.08(2)	0.11(4)	0.19(7)	0.05(4)	0.02(4)	0.04(3)
N2	0.026(14)	0.08(3)	0.08(3)	0.04(2)	-0.005(15)	0.008(16)
C17	0.10(3)	0.20(7)	0.16(5)	0.11(5)	0.03(3)	0.06(4)
C18	0.09(3)	0.20(7)	0.19(6)	0.05(5)	0.02(3)	0.06(3)
C19	0.09(2)	0.12(4)	0.16(6)	0.05(4)	0.03(3)	0.05(3)
C20	0.07(2)	0.12(4)	0.12(5)	0.04(3)	0.00(3)	0.02(2)
C21	0.06(2)	0.18(5)	0.12(5)	0.05(4)	-0.02(3)	0.03(3)
C22	0.10(4)	0.18(6)	0.22(7)	0.07(5)	0.10(5)	0.05(3)
C23	0.13(4)	0.17(6)	0.20(7)	0.09(5)	0.06(5)	0.04(4)

C24	0.15(4)	0.17(5)	0.20(7)	0.11(5)	0.06(5)	0.08(4)
N3	0.10(3)	0.20(6)	0.05(3)	0.05(3)	0.02(2)	0.04(4)
C25	0.09(3)	0.14(4)	0.09(4)	-0.01(4)	0.02(3)	-0.01(3)
C26	0.09(2)	0.15(4)	0.19(6)	0.04(5)	0.05(3)	0.04(3)
**Label**	U^{11}	U^{22}	U^{33}	U^{23}	U^{13}	U^{12}
C27	0.08(2)	0.11(4)	0.13(4)	0.07(4)	0.03(2)	0.04(3)
C28	0.11(3)	0.18(6)	0.13(4)	0.09(5)	0.03(3)	0.05(4)
C29	0.09(2)	0.13(5)	0.11(4)	-0.02(3)	0.00(3)	0.02(3)
C30	0.09(4)	0.28(9)	0.14(5)	0.01(5)	0.07(3)	0.05(5)
C31	0.16(4)	0.14(6)	0.16(5)	0.09(5)	0.09(4)	0.05(5)
C32	0.13(3)	0.17(6)	0.06(3)	0.02(3)	0.03(3)	0.05(4)
N4	0.10(3)	0.09(3)	0.09(3)	0.00(3)	0.03(3)	0.04(2)
C33	0.039(16)	0.10(3)	0.06(3)	0.03(2)	0.008(18)	0.025(19)
C34	0.076(19)	0.09(3)	0.08(3)	0.01(2)	0.02(2)	0.04(2)
C35	0.080(19)	0.10(3)	0.08(2)	0.03(2)	0.014(16)	0.07(2)
C36	0.12(3)	0.17(6)	0.10(3)	0.09(4)	-0.02(3)	0.02(4)
C37	0.11(3)	0.19(7)	0.15(4)	0.06(5)	-0.03(3)	0.04(4)
C38	0.06(3)	0.12(5)	0.17(5)	0.04(4)	0.00(2)	0.06(3)
C39	0.06(2)	0.15(5)	0.14(3)	0.02(3)	0.03(2)	0.06(3)
C40	0.066(19)	0.14(5)	0.09(3)	0.04(3)	0.013(18)	0.06(3)
N5	0.051(16)	0.09(3)	0.05(2)	0.015(18)	-0.005(15)	0.031(18)
C41	0.08(2)	0.10(3)	0.11(4)	0.06(3)	0.01(3)	0.02(2)
C42	0.07(2)	0.17(4)	0.08(4)	0.01(3)	0.01(2)	0.02(3)
C43	0.080(19)	0.11(4)	0.05(2)	0.01(2)	0.014(16)	0.02(2)
C44	0.07(2)	0.09(3)	0.09(3)	0.02(3)	0.005(19)	0.01(3)
C45	0.07(2)	0.10(4)	0.15(4)	0.07(4)	0.01(3)	0.02(3)
C46	0.10(3)	0.07(4)	0.13(4)	0.01(3)	0.06(3)	0.01(3)
C47	0.15(4)	0.20(7)	0.08(4)	0.05(4)	0.08(3)	0.06(5)
C48	0.14(3)	0.12(5)	0.05(3)	0.01(3)	0.04(3)	0.02(4)
N6	0.08(2)	0.07(3)	0.15(5)	0.04(3)	0.02(3)	0.04(2)
C49	0.07(2)	0.13(5)	0.08(4)	0.03(3)	0.00(2)	0.01(3)
C50	0.063(19)	0.13(4)	0.11(4)	0.03(3)	0.00(2)	0.02(2)
C51	0.077(19)	0.10(3)	0.09(4)	0.07(3)	0.00(2)	0.011(19)
C52	0.09(2)	0.08(3)	0.12(4)	0.02(3)	-0.02(3)	0.03(2)
C53	0.08(2)	0.12(4)	0.16(6)	0.04(4)	0.00(4)	0.03(3)
C54	0.06(3)	0.08(3)	0.23(7)	0.00(4)	0.04(4)	0.03(2)
C55	0.11(3)	0.12(4)	0.15(6)	0.10(4)	0.01(4)	-0.01(2)
C56	0.09(3)	0.08(3)	0.17(6)	0.05(4)	0.02(4)	0.04(2)
N7	0.065(19)	0.12(4)	0.05(2)	0.04(2)	0.007(18)	0.01(2)
C57	0.12(4)	0.15(7)	0.31(9)	-0.01(6)	-0.07(5)	0.02(4)
C58	0.07(2)	0.19(7)	0.34(8)	0.11(6)	-0.04(3)	0.02(3)
C59	0.10(2)	0.10(5)	0.12(3)	0.03(4)	0.00(2)	-0.02(3)
C60	0.13(3)	0.17(6)	0.10(3)	0.04(4)	-0.01(2)	0.04(4)
C61	0.12(3)	0.21(7)	0.12(3)	0.06(5)	0.02(3)	0.07(4)
C62	0.07(3)	0.08(4)	0.13(4)	0.05(3)	0.00(2)	-0.01(3)
C63	0.11(3)	0.08(4)	0.11(3)	0.02(3)	0.00(2)	0.03(3)

C64	0.10(3)	0.12(5)	0.11(3)	0.07(3)	0.04(2)	0.03(3)
N8	0.08(2)	0.06(3)	0.13(4)	0.01(2)	-0.01(2)	0.05(2)
Bi1	0.0383(8)	0.0453(12)	0.0504(15)	0.0117(10)	0.0112(8)	0.0145(8)
Br1	0.040(3)	0.047(4)	0.080(5)	0.004(3)	-0.003(3)	0.005(3)
**Label**	U^{11}	U^{22}	U^{33}	U^{23}	U^{13}	U^{12}
Br2	0.045(3)	0.065(4)	0.078(5)	0.014(4)	0.031(3)	0.023(3)
Br3	0.061(4)	0.052(4)	0.075(5)	-0.002(4)	0.024(4)	-0.002(3)
Br4	0.061(4)	0.099(6)	0.061(5)	0.006(4)	0.034(4)	0.022(4)
Br5	0.097(6)	0.128(7)	0.119(7)	0.075(5)	-0.012(5)	0.035(5)
Br6	0.037(3)	0.077(4)	0.057(4)	0.017(3)	0.006(3)	0.023(3)
Bi2	0.0669(13)	0.0650(16)	0.0432(15)	0.0075(11)	0.0058(11)	0.0305(12)
Br7	0.099(5)	0.057(5)	0.069(5)	0.000(4)	-0.001(4)	0.017(4)
Br8	0.079(4)	0.077(5)	0.064(5)	0.008(4)	0.020(4)	0.027(4)
Br9	0.067(4)	0.067(5)	0.070(5)	-0.002(4)	-0.005(4)	0.014(4)
Br10	0.080(5)	0.104(6)	0.069(5)	0.022(4)	0.028(4)	0.049(4)
Br11	0.065(4)	0.069(4)	0.065(5)	0.016(3)	0.002(3)	0.032(3)
Br12	0.133(8)	0.220(12)	0.138(9)	0.071(8)	0.000(6)	0.119(8)
Br13	0.095(5)	0.079(6)	0.095(6)	0.027(5)	0.022(5)	0.027(4)
Br14	0.121(6)	0.085(6)	0.071(5)	0.024(4)	0.018(5)	0.041(5)

Table S8. Refined atomic coordinates from single crystal data for PEA_4BiI_7

No.	Label	*x*	*y*	*z*	Occ.	*U*
1	Bi1	0.50000	0.50000	0.00000	1.000	0.060
2	I1	0.64103	0.75589	0.06947	1.000	0.084
3	I2	0.64707	0.24240	0.06700	1.000	0.079
4	I3	0.29214	0.49178	0.06497	1.000	0.104
5	I4	0.99588	0.86880	0.00266	0.500	0.115
6	C1	0.63390	-0.02880	-0.10420	1.000	0.111
7	H1A	0.54673	0.00460	-0.11602	1.000	0.134
8	H1B	0.63914	-0.13882	-0.11219	1.000	0.134
9	C2	0.72060	0.06300	-0.13260	1.000	0.136
10	H2A	0.80706	0.02435	-0.12303	1.000	0.163
11	H2B	0.72006	0.17212	-0.12307	1.000	0.163
12	C3	0.67340	0.04440	-0.19150	1.000	0.087
13	C4	0.57910	0.13730	-0.21900	1.000	0.140
14	H4	0.53798	0.20924	-0.20135	1.000	0.168
15	C5	0.54500	0.12500	-0.27250	1.000	0.176
16	H5	0.48182	0.18950	-0.29084	1.000	0.211
17	C6	0.60430	0.01650	-0.29840	1.000	0.124
18	H6	0.58445	0.01090	-0.33459	1.000	0.149
19	C7	0.69320	-0.08400	-0.27100	1.000	0.126
20	H7	0.73152	-0.15936	-0.28837	1.000	0.151
21	C8	0.72470	-0.07110	-0.21750	1.000	0.115
22	H8	0.78152	-0.14184	-0.19876	1.000	0.139

23	N1	0.66970	-0.00600	-0.04750	1.000	0.110
24	H1C	0.61759	-0.06077	-0.03164	1.000	0.165
25	H1D	0.74927	-0.03804	-0.03654	1.000	0.165
26	H1E	0.66371	0.09475	-0.04009	1.000	0.165
**No.**	Label	***x***	***y***	***z***	**Occ.**	***U***
27	C9	0.96160	0.53130	0.12450	1.000	0.133
28	H9A	0.88997	0.56699	0.13923	1.000	0.160
29	H9B	1.02437	0.61409	0.12837	1.000	0.160
30	C10	1.01910	0.38630	0.15320	1.000	0.124
31	H10A	1.08793	0.34712	0.13751	1.000	0.149
32	H10B	0.95513	0.30529	0.15127	1.000	0.149
33	C11	1.06950	0.43070	0.21040	1.000	0.107
34	C12	1.18420	0.50470	0.22570	1.000	0.144
35	H12	1.22989	0.53655	0.20066	1.000	0.173
36	C13	1.23300	0.53300	0.27770	1.000	0.194
37	H13	1.31103	0.58149	0.28793	1.000	0.231
38	C14	1.16220	0.48600	0.31410	1.000	0.178
39	H14	1.19493	0.50164	0.34927	1.000	0.213
40	C15	1.04420	0.41800	0.29940	1.000	0.153
41	H15	0.99568	0.39239	0.32418	1.000	0.182
42	C16	0.99960	0.38810	0.24730	1.000	0.116
43	H16	0.92159	0.33867	0.23696	1.000	0.139
44	N2	0.91880	0.49400	0.06870	1.000	0.118
45	H2C	0.88508	0.57863	0.05183	1.000	0.177
46	H2D	0.98492	0.46214	0.05537	1.000	0.177
47	H2E	0.86067	0.41860	0.06540	1.000	0.177

Table S9. Anisotropic thermal displacement parameters from single crystal data for PEA_4BiI_7

**Label**	U^{11}	U^{22}	U^{33}	U^{23}	U^{13}	U^{12}
Bi1	0.0837(4)	0.0414(3)	0.0523(3)	0.0000(2)	0.0041(2)	0.0077(3)
I1	0.1185(8)	0.0553(4)	0.0648(5)	-0.0132(3)	-0.0165(5)	0.0097(4)
I2	0.1083(7)	0.0543(4)	0.0633(5)	0.0131(3)	-0.0081(4)	0.0046(4)
I3	0.1385(10)	0.1082(8)	0.0710(6)	0.0028(5)	0.0316(6)	0.0193(6)
I4	0.0667(11)	0.148(2)	0.1219(17)	0.0073(13)	-0.0001(11)	0.0002(12)
C1	0.140(15)	0.132(13)	0.068(6)	0.002(8)	0.035(8)	0.002(10)
C2	0.166(17)	0.166(15)	0.079(7)	-0.005(8)	0.034(8)	-0.044(13)
C3	0.111(11)	0.087(8)	0.071(6)	0.004(5)	0.036(6)	0.001(7)
C4	0.180(19)	0.123(14)	0.124(9)	0.024(11)	0.046(11)	0.069(12)
C5	0.20(2)	0.19(2)	0.127(11)	0.062(14)	0.006(14)	0.060(16)
C6	0.138(16)	0.168(17)	0.064(8)	0.011(8)	0.011(7)	-0.069(10)
C7	0.146(16)	0.146(15)	0.103(9)	-0.034(10)	0.063(10)	-0.013(10)
C8	0.131(14)	0.119(12)	0.098(8)	0.004(9)	0.026(9)	0.040(10)
N1	0.088(8)	0.189(15)	0.055(5)	-0.003(7)	0.019(5)	0.011(8)
C9	0.084(11)	0.176(15)	0.124(10)	0.000(10)	-0.019(10)	0.016(11)

C10	0.147(16)	0.117(12)	0.103(7)	-0.003(8)	0.013(8)	-0.019(10)
C11	0.079(9)	0.125(12)	0.106(7)	-0.016(9)	-0.014(6)	0.009(8)
C12	0.103(11)	0.138(17)	0.167(14)	0.011(11)	-0.037(10)	-0.005(9)
C13	0.19(2)	0.15(2)	0.192(18)	-0.009(19)	-0.094(15)	-0.016(15)
**Label**	U^{11}	U^{22}	U^{33}	U^{23}	U^{13}	U^{12}
C14	0.24(2)	0.124(18)	0.120(12)	-0.009(12)	-0.093(12)	0.052(14)
C15	0.22(2)	0.125(15)	0.097(8)	-0.004(11)	-0.012(10)	0.053(14)
C16	0.131(13)	0.110(13)	0.100(8)	-0.018(9)	0.002(8)	0.006(9)
N2	0.093(10)	0.161(15)	0.101(8)	0.010(7)	0.021(7)	0.018(8)
Bi1	0.0837(4)	0.0414(3)	0.0523(3)	0.0000(2)	0.0041(2)	0.0077(3)

References:

(1) Valastro, S.; Gavranovic, S.; Deretzis, I.; Vala, M.; Smecca, E.; La Magna, A.; Alberti, A.; Castkova, K.; Mannino, G. Temperature-Dependent Excitonic Band Gap in Lead-Free Bismuth Halide Low-Dimensional Perovskite Single Crystals. *Advanced Optical Materials* **2024**, *12* (11), 2302397. https://doi.org/10.1002/adom.202302397.
